# Characterization of a small-molecule inhibitor targeting NEMO/IKKβ to suppress colorectal cancer growth

**DOI:** 10.1038/s41392-022-00888-1

**Published:** 2022-03-09

**Authors:** Zhenlong Yu, Jian Gao, Xiaolei Zhang, Yulin Peng, Wenlong Wei, Jianrong Xu, Zhenwei Li, Chao Wang, Meirong Zhou, Xiangge Tian, Lei Feng, Xiaokui Huo, Min Liu, Mingliang Ye, De-an Guo, Xiaochi Ma

**Affiliations:** 1 Pharmaceutical Research Center, Second Affiliated Hospital, Dalian Medical University, Dalian, 116000 China; 2 College of Pharmacy, College (Institute) of Integrative Medicine, Dalian Medical University, Dalian, 116044 China; 3 Jiangsu Key Laboratory of New Drug Research and Clinical Pharmacy, Xuzhou Medical University, Xuzhou, 221004 China; 4 CAS Key Laboratory of Separation Sciences for Analytical Chemistry, National Chromatographic R&A Center, Dalian Institute of Chemical Physics, Chinese Academy of Sciences, Dalian, 116023 China; 5 Shanghai Research Center for Modernization of Traditional Chinese Medicine, National Engineering Research Center for TCM Standardization Technology, Shanghai Institute of Materia Medica, Chinese Academy of Sciences, Shanghai, 201203 China; 6 Academy of Integrative Medicine, Shanghai University of Traditional Chinese Medicine, 1200 Cailun Road, Shanghai, 201203 China; 7 Neurology Department, Dalian University Affiliated Xinhua Hospital, Dalian, 116021 China

**Keywords:** Drug development, Target identification

## Abstract

NEMO/IKKβ complex is a central regulator of NF-κB signaling pathway, its dissociation has been considered to be an attractive therapeutic target. Herein, using a combined strategy of molecular pharmacological phenotyping, proteomics and bioinformatics analysis, Shikonin (SHK) is identified as a potential inhibitor of the IKKβ/NEMO complex. It destabilizes IKKβ/NEMO complex with IC_50_ of 174 nM, thereby significantly impairing the proliferation of colorectal cancer cells by suppressing the NF-κB pathway in vitro and in vivo. In addition, we also elucidated the potential target sites of SHK in the NEMO/IKKβ complex. Our study provides some new insights for the development of potent small-molecule PPI inhibitors.

## Introduction

Nuclear factor-κB (NF-κB) denotes a family of inducible transcription factors that regulate multiple important physiological and pathological cellular processes.^1^ Several studies have indicated that NF-κB is excessively or inappropriately activated in a variety of pathological cellular processes, especially inflammation and cancer.^2–5^ NF‑κB activation occurs via two major signaling pathways: the canonical and the non‑canonical NF‑κB signaling pathways. The activation of the canonical NF‑κB pathway depends on the formation of the IκB kinase (IKK) complex, which is composed of three subunits: the catalytic subunits IKKα and IKKβ, and the regulatory subunit NF-κB essential modulator (NEMO or IKKγ).^6,7^ In resting cells, canonical NF-κB protein usually binds to its inhibitor IκBα and resides in an inactive state in the cytoplasm. Under stimuli such as inflammatory cytokines (TNF-α and IL-6), IκBα is phosphorylated by IKK complex, and then occurs ubiquitination and proteasomal degradation, leading to the release of free NF-κB dimer (containing the p50 and p65 subunits).^8^ The dimer then translocates into the nucleus and activates the transcription of downstream target genes. Aberrant activation of canonical NF-κB induces the expression of multiple genes, including several inflammatory response mediators such as chemokines (CCL5 and CXCL8), cytokines (TNF-α and IL-6), and adhesion molecules (VCAM and ELAM), in addition to functional enzymes such as inducible nitric oxide synthase (iNOS) and cyclooxygenase-2 (COX-2).^9,10^ Enhanced NF-κB signaling has been observed in many chronic inflammatory conditions such as inflammatory bowel disease,^11^ colitis^11^, and hepatitis,^12^ which greatly increase the risks of cancer occurrence.^2,13^ The non‑canonical NF-κB signaling pathway is required for the regulation of lymphoid organ development, B cell survival and maturation, and the differentiation of osteoclasts.^14,15^ Unlike canonical pathways, its activation is characteristically slow and persistent, and mediated by a different set of cell surface receptors. Most of these receptors also stimulate the canonical NF‑κB pathway through IKK complex^16,17^ and mediate biological processes. Therefore, the IKK complex is a very attractive therapeutic target, and inhibition of its formation represents an attractive anti-inflammatory and anticancer strategy, without affecting the basal NF-κB activity required for normal B and T cell function.

Previous studies have indicated that IKKβ kinase is vital for the activation of the NF-κB signaling pathway triggered by inflammatory stimuli via the degradation of the IκBα subunit,^8,18^ which is critical for the production of pro-inflammatory cytokines and the development of various malignancies, including breast cancer, colorectal cancer and liver cancer.^19–21^ The binding of the regulatory subunit NEMO to the C-terminal region of catalytic subunit IKKβ (termed the NEMO binding domain, NBD),^22,23^ commonly leads to conformational changes of IKKβ that expose its phosphorylation sites at S177 and S181,^24^ thereby the promoting phosphorylation and degradation of IκB proteins to activate NF-κB. Therefore, inhibition of NEMO/IKKβ complex formation or decreasing its stability would be a promising therapeutic target for the treatment of inflammatory and malignant diseases.

Due to its importance in pathogenesis, the NEMO/IKKβ complex has become a focus of research in recent years, and screening of potential inhibitors based on the vital interaction between NEMO and IKKβ protein has also received increasing attention. The crystal structure of the NEMO/IKKβ complex^23^ revealed the molecular details of their interaction, and provides an excellent platform for the discovery of inhibitors. Briefly, the complex is a 4-helix bundle consisting of NEMO and IKKβ domains each consisting of two chains. Two NEMO chains form a homodimer, while each IKKβ chain is loosely bound to each NEMO chain at the N terminus, and tightly wedged between the NEMO dimer at the C terminus. The interaction interface of NEMO and IKKβ involves the NBD (L737–L742) of IKKβ and residues S85-Q101 of NEMO. Among them, D738, W739, and W741 of IKKβ were identified as critical hotspots for formation of the NEMO/IKKβ complex.^22,23,25^

The inhibitors of NEMO/IKKβ complex formation discovered to date are mainly poly- or oligo-peptides. For example, a 11-residue NBD-containing peptide was found to inhibit the formation of the NEMO/IKKβ complex (IC_50_~200 μM)^22^ by binding to the NBD-binding site of NEMO, further inhibiting cytokine-induced NF-κB activation and NF-κB-dependent gene expression.^22,26–28^ However, the low potency, poor bioavailability and bio-stability (e.g., serum half-life of ∼15 min) of these polypeptide inhibitors in vitro and in vivo, greatly limited their clinical application prospects. To avoid these shortcomings, potential small-molecule inhibitors were developed by virtual screening based on the crystal structure of the NEMO/IKKβ complex. However, no potent small-molecule inhibitor has been reported to effectively disrupt the NEMO/IKKβ complex in vivo, although some small-molecule inhibitors with modest potency against the NEMO-IKK interaction (IC_50_∼ 20 μM) have been reported.^29–31^ However, the lack of a clear inhibitory mechanism halted their development and clinical application. Therefore, it is urgent to develop potent small-molecule PPI inhibitors that can disrupt the critical NEMO/IKKβ interaction both in vitro and in vivo.

Due to their beneficial pharmacological activities and low toxicity, natural products are important in cancer chemotherapy. The classical naphthoquinone natural product shikonin (SHK) is a bioactive constituent of *Lithospermum erythrorhizon*, which has a long history of more than 2000 years of use as a Chinese medicinal herb known as *zicao* (紫草).^32^ Previous studies have suggested that SHK possesses various physiological and pharmacological properties, including anti-inflammatory, wound healing, and anti-cancer effects.^32–35^ Recent studies have demonstrated that SHK has significant anti-tumor activity, such as inducing cell cycle arrest and apoptosis, inhibiting the proliferation of various types of cancer cells.^36–38^ The anti-tumor mechanism of SHK is mediated by the Bcl-2 family proteins and the NF-κB pathway.^39,40^ However, the mechanism underlying its effects against colon cancer has not yet been characterized.

To our best knowledge, this is the first study to unequivocally identify the NEMO/IKKβ complex as the anti-tumor target of SHK using a combination strategy of molecular pharmacological phenotyping, proteomics, and bioinformatics analysis. SHK was found to be the most active small-molecule PPI inhibitor of NEMO/IKKβ complex (IC_50_~170 nM) reported so far. It effectively blocks the complex formation in vitro, in cell culture, and even in animal models, reversing a number of colon cancer cell phenotypes such as proliferation, migration, and invasiveness. We used various chemo-biological approaches, including hydrogen–deuterium exchange mass spectrometry (HDX-MS), binding assays with different mutants and molecular dynamics (MD) simulations, to elucidate the key action sites and reveal the novel inhibition mode of SHK against the NEMO/IKKβ complex. Taken together, these results revealed the antitumor mechanism of SHK, and provide an advanced understanding of NEMO/IKKβ complex function, all of which providing a useful guide for the development of potent small-molecule PPI inhibitors of NEMO/IKKβ.

## Results

### SHK suppresses the malignant phenotypes of colorectal cancer cells in vitro and in vivo

**Fig. 1:**
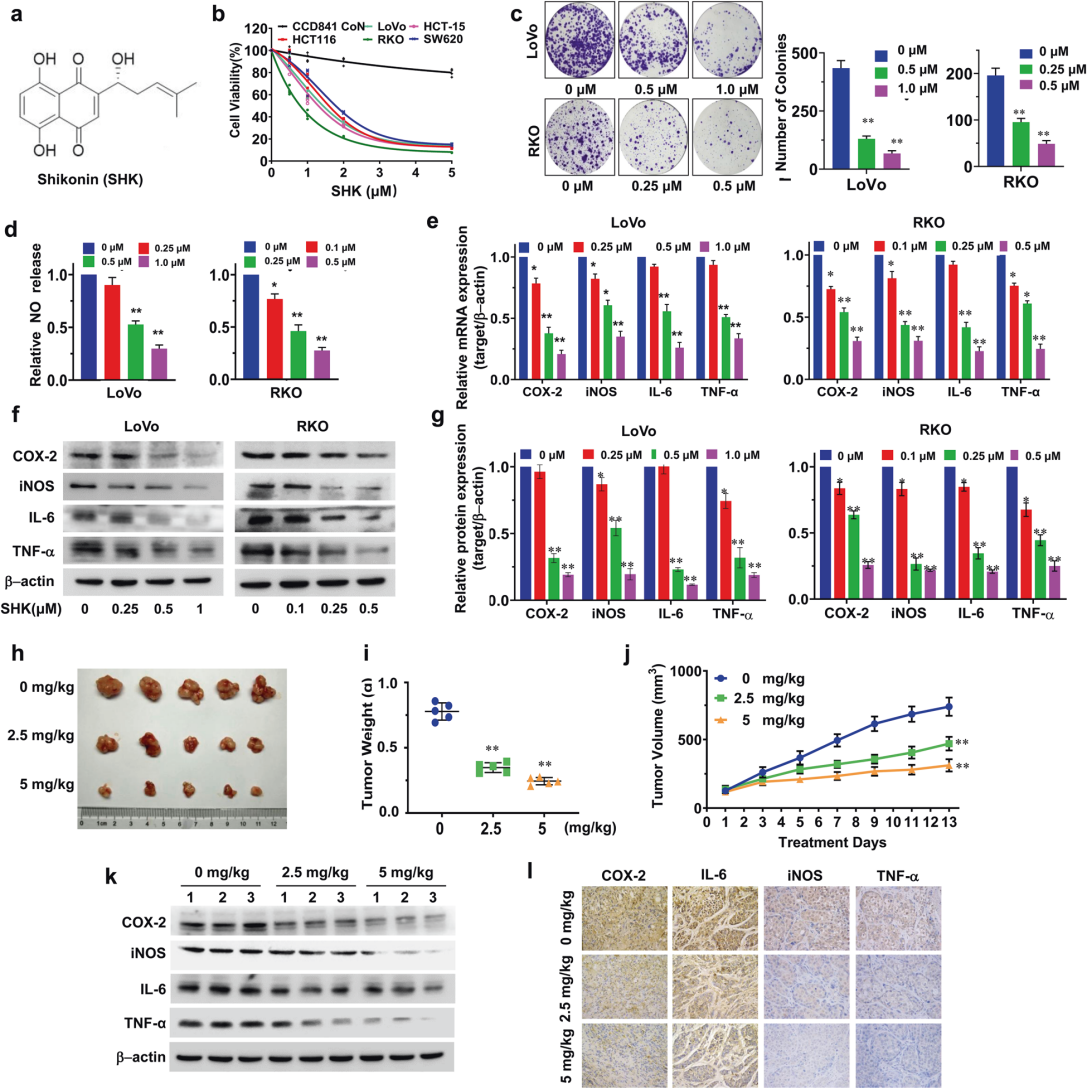
Effect of shikonin on the malignant phenotypes of human colorectal cancer cells. **a** Chemical structure of shikonin (SHK). **b** Colorectal cancer cells (LoVo, RKO, HCT-15, HCT-116, and SW620) and normal human colonic epithelial cells (CCD841 CoN) were cultured with the indicated concentrations of SHK for 48 h, after which the cell viability was determined using the CCK-8 assay. **c** The SHK-induced reduction of colony formation was analyzed, and the colony formation numbers were calculated. **d** The NO content following SHK treatment was determined using the Griess reagent. LoVo and RKO cells were cultured with SHK. The expression of inflammatory markers (COX-2, iNOS, IL-6, and TNF-α) was analyzed by qPCR (**e**) and western blotting (**f**). A quantitative analysis of these proteins was also performed (**g**). The data are presented as the means ± SD of at least three separate experiments. (*p < 0.05, **p < 0.01, SHK treatment group vs. vehicle control group). In vivo antitumor efficacy of SHK in a LoVo xenograft tumor model. Tumor appearance (**h**), total tumor weights (**i**) and tumor volumes (**j**) were assessed. The expression of inflammatory markers (COX-2, iNOS, IL-6, and TNF-α) in tumor tissues was analyzed by western blotting (**k**) and immunohistochemical analysis (**l**). Data were presented as the means ± SD from *n* = 5 mice/group. (*p < 0.05, **p < 0.01, SHK treatment group vs. vehicle control group. Magnification, ×200)

The anti-cancer effects of SHK (Fig. 1a) were first evaluated by treating colorectal cancer cells with various concentrations of SHK (0 μM, 0.5 μM, 1 μM, 2 μM, and 5 μM). It was found that treatment with SHK resulted in dose-dependent growth inhibition of various cancer cell lines (LoVo, RKO, SW620, HCT15, and HCT116 cells). Moreover, this inhibitory effect was absent in normal human colonic epithelial cells (CCD841 Con cells) (Fig. 1b and Supplementary Fig. 1a). As SHK was observed to most significantly inhibit the proliferation in LoVo and RKO cells, these two cell lines were used for further mechanistic studies. SHK treatment significantly resulted in colony formation suppression (Fig.1c), a remarkable reduction of CFSE dilution, and a great increase of G0/G1 phase and apoptotic cells, which were consistent with cell proliferation inhibition (Supplementary Fig. 1b and c and Supplementary Fig. 2a and c). Additionally, changes of cell morphology and lower cellular mobility were also observed following SHK treatment, and the effect was more pronounced at higher doses (Supplementary Fig. 3a–c). Furthermore, SHK treatment greatly decreased the protein levels Cyclin E1/D1, CDK4 and MMP2/9, while increasing the levels of cleaved caspases 3 and 9, cleaved PARP, and TIMP2 (Supplementary Fig. 2b and e and Supplementary Fig. 3d).

Inflammation is a hallmark of cancer that substantially contributes to the development and progression of malignancies.^41^ Our results showed that SHK dramatically decreased the release of NO (Fig. 1d), and suppressed the expression of inflammatory markers such as COX-2, iNOS, IL-6, and TNF-α in a dose-dependent manner in both LoVo and RKO cells **(**Fig. 1e–g**)**. Similar inhibitory effects were also observed in chronic inflammatory models simulated by LPS (Supplementary Fig. 4), implying that SHK could block the progression of colorectal cancer in vitro by downregulating inflammatory signaling pathways.

A tumor xenograft mouse model of human colorectal cancer was used to further explore tumor growth inhibition by SHK in vivo. SHK (2.5 and 5 mg/kg daily) dramatically decreased both tumor volume and tumor weight in mice (Fig. 1h–j), and resulted in smaller nuclei and lower nuclear-cytoplasmic ratio (Supplementary Fig. 5a). Furthermore, there were also significant changes in the expression of inflammatory markers (COX-2, iNOS, TNF-α, and IL-6) (Fig. 1k and l), as well as vital proteins related to the cell cycle progression (CDK4 and Cyclin D1), apoptosis (cleaved-PARP and cleaved-Caspase3) and migration (MMP2/9) (Supplementary Fig. 5b) in SHK-treated tumors. Taken together, all these data suggested that the anti-inflammatory effect of SHK can inhibit the progression of colorectal cancer xenografts in vivo with slight side effects (Supplementary Fig. 5c).

### Identification of the molecular target of SHK

**Fig. 2:**
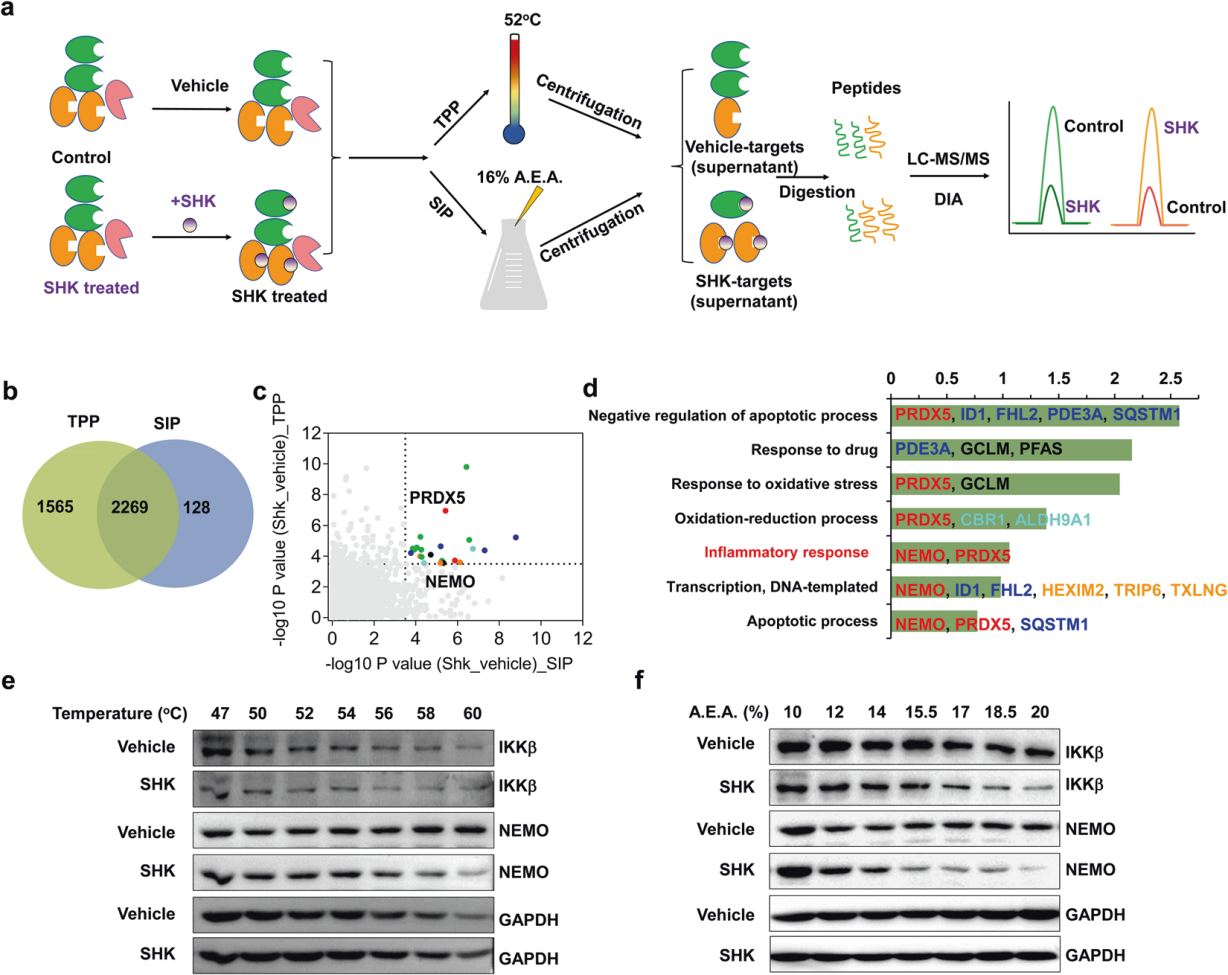
NEMO and IKKβ were identified as the potential targets of SHK by TPP and SIP assays. **a** Schematic representation of the TPP and SIP methods for screening of target proteins of SHK. **b** The Venn diagram of potential target proteins identified by the TPP and SIP methods. **c** A total of 22 candidate targets were screened by filtering the *P* value of each protein identified in the TPP and SIP assays. The *P* value was presented as −log10. Red dots indicate proteins destabilized by SHK, blue dots indicated proteins stabilized by SHK, and green dots indicate proteins were identified to be stabilized by SHK using one method, while being identified as being destabilized using the other method. **d** The GO biological process enrichment of the 22 candidates. The Y-axis lists the name of each category, the X-axis shows the −log10 (*p*-value). **e** Western blotting demonstrated that NEMO and IKKβ were dissociated when incubated with SHK by using the TPP method. **f** Western blotting demonstrated that NEMO and IKKβ were dissociated when incubated with SHK by using the SIP method

To further interrogate the mechanism through which SHK suppresses the progression of colorectal cancer, thermal proteome profiling (TPP)^42^ and solvent-induced protein precipitation (SIP)^43^ methods were employed to identify the target proteins of SHK. These two methods are based on the fact that the precipitation rate of the target proteins to temperature or solvents is likely changed after direct interaction with SHK. Consequently, interacting and free target proteins can be differentially precipitated when they are subjected to denaturation by heating or solvent treatment (Fig. 2a).

In the present study, a total of 2269 proteins were quantified using the TPP and SIP methods (Fig. 2b). After applying a cutoff of 3.5 for the −log10 *P* value in both the TPP and SIP datasets, 22 proteins were identified as the top significantly changed proteins upon SHK treatment (Fig. 2c). These proteins were candidate targets of SHK in colorectal cancer cells, as stability shifts were observed in both the heating and solvent treatments. The in vitro and in vivo experiments had indicated that SHK could suppress the progression of colorectal cancer by inhibiting inflammatory pathways. After GO biological process analysis of the 22 candidate proteins, only NEMO and PRDX5 were identified in an inflammation-related functional cluster of “Inflammatory response” (Fig. 2d). This finding was highly correlated with our experimental results mentioned above. NEMO was reported to control the pathophysiology of inflammation and carcinogenesis via NF-κB signaling.^44,45^ To verify changes of NEMO-related signaling induced by SHK on the basis of MS analysis, we used different temperatures and percentages of solvent mixtures to denature proteins in the vehicle and SHK-treated groups and analyzed the supernatants by immunoblotting. The western blot analysis revealed that SHK treatment accelerated the precipitation of NEMO protein to temperature or solvent changes in both the denaturation methods (Fig. 2e and f, middle two bands), all of which indicated that NEMO may have the direct interaction with SHK. It is well known that NEMO and IKKβ are form the IKK complex,^7,23^ and similarly, the effects of SHK on IKKβ was also explored. It should be noted that IKKβ was not identified among the overlapping proteins, possibly due to its low expression. As expected, we found that IKKβ may have direct interaction with SHK according to western blot analysis (Fig. 2e and f, first two bands). Our experiments further confirmed that an unrelated protein such as glyceraldehyde 3-phosphate dehydrogenase (GAPDH) was not affected by SHK (Fig. 2e and f, bottom two bands). These results demonstrated that the NEMO/IKKΒ complex would be the potential target of SHK in downregulating the NF-κB pathway. In addition, PRDX5 was also enriched in the identified inflammation-related functional cluster. PRDX5 is a binding partner of NRF-2 that can promote its activation to induce the expression of a group of functionally diverse cytoprotective factors such as heme oxygenase-1 (HO1) to attenuate inflammation.^46,47^ However, SHK did not alter the protein levels of NRF-2 and HO1, and the translocation of NRF-2 into the nucleus (Supplementary Fig. 6a–c), which indicated that PRDX5 was not a target protein of SHK. Besides, to exclude the possibility that NEMO’s or IKKβ’s protein stability is affected by SHK, we incubated cell lysate and SHK for various indicate time (15, 30 and 45 min), and then collected the supernatant after high-speed centrifugation. Western blotting showed that SHK did not affect the stability of NEMO or IKKβ protein (Supplementary Fig. 6d). All these results suggested that the NEMO/IKKΒ complex was the likely target through which SHK exerts its anti-cancer activity, rather than PRDX5.

### SHK Disrupts the IKKβ-NEMO interaction in cancer cells

**Fig. 3:**
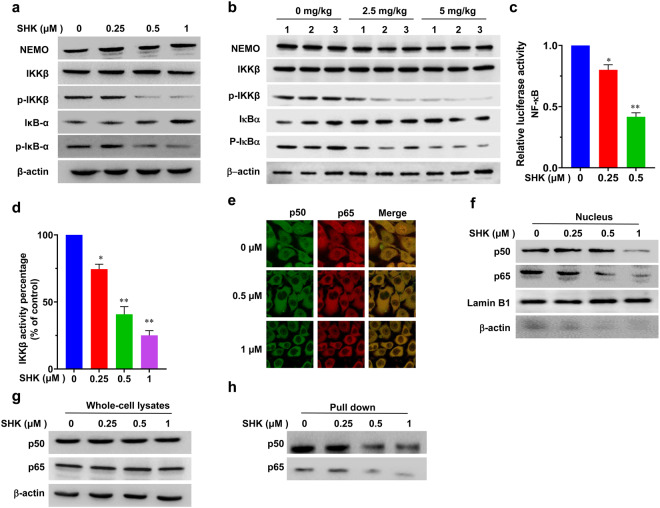
Effect of SHK on NEMO/IKKβ/NF-κB signaling. **a** The protein levels of NEMO, p-IKKβ/IKKβ, and p-IκBα/IκBα in LoVo cells treated with the indicated doses of SHK were analyzed by western blotting. **b** The protein levels of NEMO, p-IKKβ/IKKβ, p-IκBα/IκBα protein in tumor tissues were analyzed by western blotting. **c** LoVo cells were co-transfected with pNFκB-luc containing a Renilla luciferase reporter (as internal control) for 24 h and then treated with SHK for 48 h. Luciferase activity was determined using a dual-luciferase reporter assay system. **d** The IKKβ kinase activity was assessed using a spectrophotometric quantitative detection kit in LoVo cells. The relative activity was calculated using the provided formula. **e** The subcellular localizations of p50/p65 in LoVo cells following 48 h of treatment with SHK was examined by confocal microscopy analysis. The protein levels of p65 and p50 in nucleus (**f**) and whole-cell lysates (**g**) were measured by western blot analysis. Lamin B1 and β-actin were used as controls for sample loading. **h** The binding of p65 and p50 to a COX-2 promoter probe was analyzed using a streptavidin-agarose pull-down assay. The data are presented as the means ± SD of at least three separate experiments. (*p < 0.05, **p < 0.01, SHK treatment group vs. vehicle control group)

NEMO/IKKΒ has been previously shown to be the major subunit responsible for the phosphorylation of IκBs,^48,49^ and then liberating free NF-κB dimers (containing the p50 and p65 subunits) to translocate into the nucleus to promote the transcription of pro-inflammatory genes.^49,50^ Our results indicated that SHK did not significantly alter their overall expression levels, and instead suppressed the phosphorylation and kinase activity of IKKβ in a dose-dependent manner. This led to decreasing protein levels of p-IκB-α (Fig. 3a–c), implying that the NEMO/IKKβ complex is responsible for the phosphorylation of IκBs, and hence a potential target of SHK. To further verify this, we determined the changes in downstream signaling after SHK treatment. As expected, a significant inhibition on NF-κB luciferase activity (Fig. 3d) and the reduced translocation of NF-κB p50/p65 subunits into the nucleus was also observed after SHK treatment (Fig. 3e and f) with no affecting on their overall protein levels (Fig. 3g). To further evaluate the effect of SHK on NF-κB binding activity, we also performed a streptavidin-agarose pull-down assay using the COX-2 promoter, which is tightly regulated by the transcription factor NF-κB.^50,51^ As the nuclear translocation of NF-κB p50/p65 subunits was inhibited by SHK, its binding to COX-2 promoter was also attenuated (Fig. 3h). Taken together, these data suggested that SHK suppressed IKKβ kinase activity to attenuate NF-κB activation, leading to significant inhibition of its binding to target gene promoters, reducing the proliferation of colorectal cancer cells. These results further confirmed that NEMO/IKKβ is a likely target of SHK based on the observed molecular biological phenotypes. To rule out the possibility that the suppression of SHK on IKKβ activity and pathway activation might be due to indirect upstream effects, we further analyzed the effects of SHK on TAK1, an upstream positive regulator of IKKβ/NEMO complex. The results showed that SHK did not change its phosphorylation level, nor its overall expression level (Supplementary Fig. 6e), which implying that SHK had no effects on the upstream regulators of IKKβ/NEMO complex. Thus, we further focused on the underlying mechanism and molecular interaction between SHK and NEMO/IKKβ.

### Characterization of the molecular interaction between SHK and NEMO/IKKβ

**Fig. 4:**
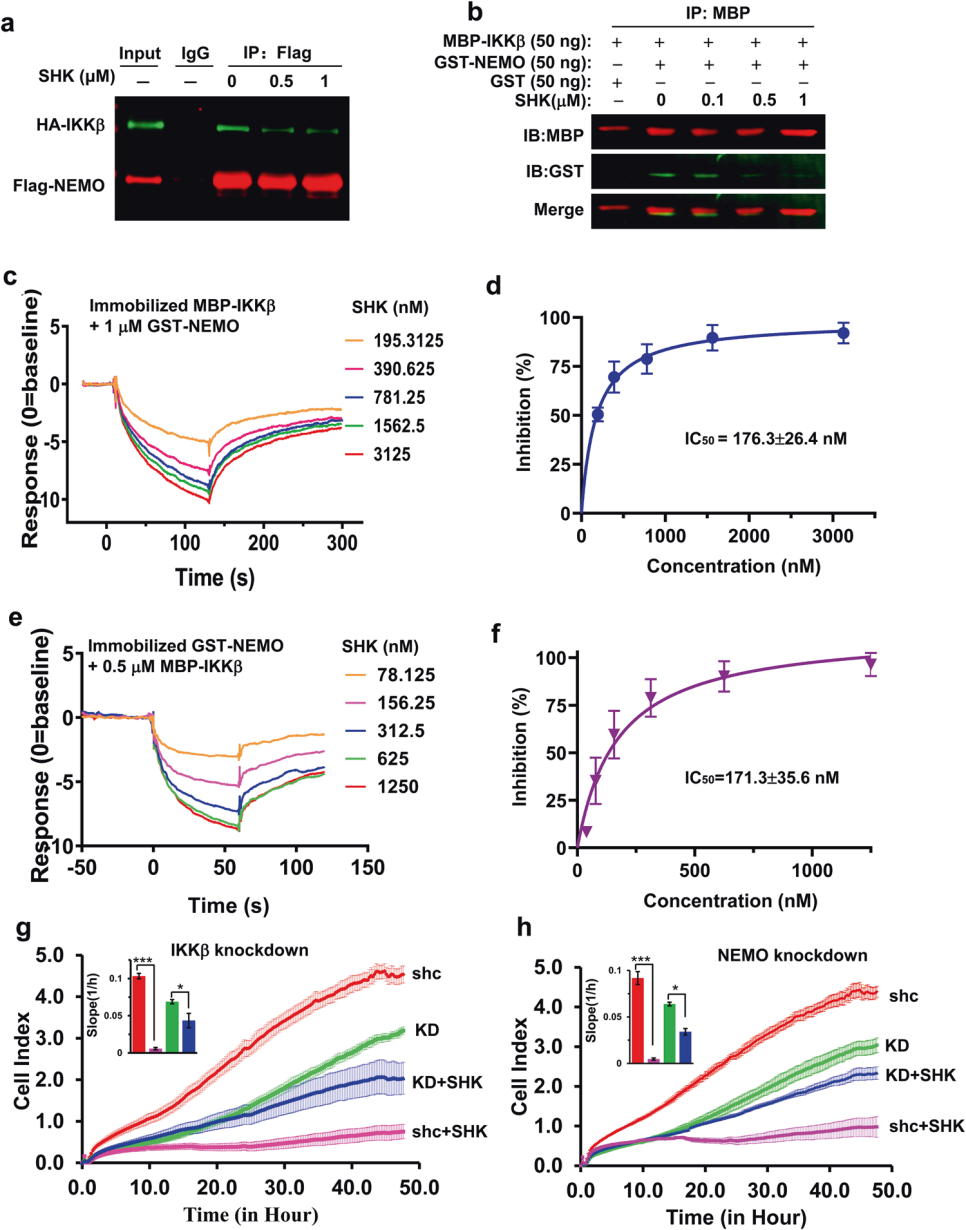
Effect of SHK on the NEMO/IKKβ complex. **a** HEK293T cells were co-transfected with eukaryotic plasmids expressing full-length Flag-NEMO and HA-IKKβ, and then treated with SHK. Whole-cell extracts were collected and subjected to a Co-IP assay and western blotting using antibodies against Flag and HA. **b** Prokaryotic plasmids were used to express truncated MBP-IKKβ (701–745) and GST-NEMO (1–196) in *E. coli*. SHK was pre-incubated with IKKβ and NEMO, respectively. The interaction of NEMO/IKKβ was analyzed by Co-IP and western blotting using antibodies against MBP and GST. **c** SHK was pre-incubated with 1 μM GST-NEMO for 30 min, and injected to determine the binding of GST-NEMO to MBP- IKKβ on the chip surface. **d** The inhibitory effects of SHK were quantified to calculate the IC_50_. **e** SHK was pre-incubated with 0.5 μM MBP- IKKβ for 30 min, and injected to determine the binding of MBP- IKKβ to GST-NEMO on the chip surface. **f** The inhibitory effects of SHK were quantified to calculate the IC_50_. **g**, **h** After stable transfected with IKKβ (**g**) or NEMO (**h**) shRNA or nonspecific control shRNA with lentivirus infection, LoVo cells were incubated with or without SHK (1 μM). Cell index curves were generated in real time using an xCELLigence instrument

We hypothesized that SHK might hinder this molecular interaction between IKKβ and NEMO, thus inhibiting IKKβ kinase activity. We first constructed eukaryotic plasmids encoding full-length HA-IKKβ and Flag-NEMO, as well as prokaryotic plasmids encoding truncated MBP-IKKβ (701–745) and GST-NEMO (1–196).^23^ Co-immunoprecipitation (Co-IP) experiments revealed that exogenously expressed full-length HA-IKKβ and Flag-NEMO could form a stable complex, and their association was greatly reduced by the addition of SHK (Fig. 4a). Additionally, pretreatment with SHK also blocked the critical association of MBP-IKKβ (701–745) and GST-NEMO (1–196) peptides in vitro (Fig. 4b), demonstrating that SHK dramatically impaired NEMO/IKKβ complex formation, and that the corresponding ligand-binding pocket is most likely located between residues 701–745 of IKKβ and 1–196 of NEMO.

To elucidate the molecular interaction between SHK and NEMO/IKKβ, we performed surface plasmon resonance (SPR) and microscale thermophoresis (MST) assays using MBP-IKKβ (701–745) and GST-NEMO (1–196) peptides. First, we immobilized MBP-IKKβ (701–745) on the chip surface to determine the binding affinity of GST-NEMO (1–196) using the SPR assay. This binding affinity of NEMO/IKKβ is consistent with literature reports, with a *K*_D_ value of 260.73 ± 62.03 nM (Supplementary Fig. 7a). Similarly, when immobilized GST-NEMO (1–196) peptide, direct binding affinity of MBP-IKKβ (701–745) was also observed with *K*_D_ value of 390.03 ± 116.66 nM (Supplementary Fig. 7b). Consistent results were also obtained in the MST assay, with a *K*_D_ value of 209.4 ± 32.5 nM for GST-NEMO (1–196), and 153.4 ± 19.3 nM for MBP-IKKβ (701–745) (Supplementary Fig. 7c and d). The two analysis methods confirmed that there is a strong affinity between MBP-IKKβ (701–745) and GST-NEMO (1–196) peptides.

However, SHK exhibited slight affinity for MBP-IKKβ (701–745) (**K**_D_ = 8.19±1.92 μM and 15.2 ± 4.0 μM) and GST-NEMO (1–196), with *K*_D_ values of 8.29 ± 2.38 μM and 14.2 ± 1.70 μM according to the SPR and MST assays, respectively (Supplementary Fig. 7e-h). Notably, the *K*_D_ value was much higher than the IC_50_ value (0.6–2.5 μM) for the inhibition of proliferation in colorectal cancer cells (Supplementary Fig. 1a). Furthermore, we performed the SPR assay to evaluate the effect of SHK on the binding affinity of MBP-IKKβ (701–745) and GST-NEMO (1–196). Interestingly, SHK could readily impair the binding of GST-NEMO (1–196) to MBP-IKKβ (701–745), and its IC_50_ value was 176.3 ± 26.4 nM (Fig. 4c, d). Consistent results were also observed with immobilized GST-NEMO (1–196), and the IC_50_ value of SHK for inhibiting the binding of MBP-IKKβ (701–745) to GST-NEMO (1–196) was 171.3 ± 35.6 nM (Fig. 4e, f). These data demonstrated that SHK had weak affinity for IKKβ or NEMO, but it exhibited an extraordinary ability to attenuate the association of the NEMO/IKKβ complex at the nanomolar level. To our best knowledge, SHK is the first highly potent small-molecule PPI inhibitor of NEMO/IKKβ complex formation. Furthermore, an in vitro fluorescence resonance energy transfer (FRET) assay was performed to directly evaluate the PPI inhibition activity of SHK. As shown in Supplementary Fig. 7i, SHK effectively impaired NEMO/IKKβ interaction with IC_50_ values of 231.03 ± 9.39 nM.

To further illustrate that the anti-tumor effect of SHK is caused by the inhibition of NEMO/IKKβ complex formation, we detected the inhibitory effect of SHK on IKKβ or NEMO depletion LoVo cells in real time using the xCELLigence instrument. IKKβ or NEMO depletion did inhibit the signaling pathway, resulting in significantly lower growth rate of knockdown cells than that of wildtype cells. In IKKβ or NEMO depletion cells by lentivirus, the inhibitory effects of SHK were significantly weakened (Fig. 4g and h). These results demonstrated that NEMO/IKKβ was the potential molecular target of SHK and mediated its antitumor effect.

### Conformational dynamics of the NEMO/IKKβ complex during SHK binding

**Fig. 5:**
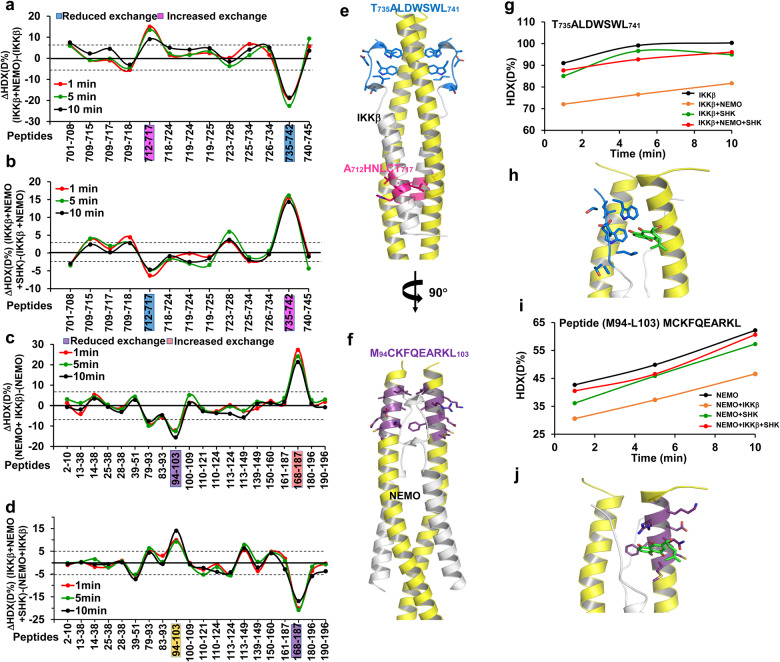
Effect of SHK on the conformational dynamics of NEMO/IKKβ. a Comparison of the HDX of IKKβ in the presence and absence of NEMO for the 13 peptides identified from IKKβ over the measured time points (red-1 min; green-5 min; black-10 min) (pink: Increased exchange, blue: decreased exchange). Positive and negative values indicate decreased or increased HDX, respectively, when IKKβ was incubated in the presence of NEMO or alone. Differences > ±7.5% are considered significant. **b** Comparison of the HDX of IKKβ in the presence of NEMO/SHK and NEMO only for the 13 peptides identified from IKKβ over the measured time points (red-1 min; green-5 min; black-10 min) (pink: Increased exchange, blue: decreased exchange). Differences > ±4.0% are considered significant. **c** Comparison of the HDX of NEMO in the presence and absence of IKKβ for the 21 peptides identified from NEMO over the measured time points (red-1 min; green-5 min; black-10 min) (pink: Increased exchange, blue: decreased exchange). Positive and negative values indicate decreased or increased HDX, respectively, when NEMO was incubated in the presence of IKKβ or alone. Differences > ±7.5% are considered significant. **d** Comparison of the HDX of NEMO in the presence of IKKβ/SHK to IKKβ only for the 21 peptides identified from NEMO over the measured time points (red-1 min; green-5 min; black-10 min). (yellow: Increased exchange, blue: decreased exchange). Differences > ±4.0% are considered significant. **e** Regions showing significant changes of 712–715 (pink) and 735–742 (blue) of IKKβ in dynamics (i.e., HDX) in the presence of SHK compared to IKKβ alone or NEMO/IKKβ are plotted on the crystal structure of the NEMO/IKKβ complex. **f** Regions showing significant changes of 98–103 (purple) of NEMO in dynamics (i.e., HDX) in the presence of SHK compared to NEMO alone or NEMO/IKKβ are plotted on the crystal structure of the NEMO/IKKβ complex. Regions colored gray indicate insignificant differences in HDX between experimental states or regions not covered by peptides. **g** Deuterium uptake plots for the peptide located in IKKβ (peptide 735–742, black) in the presence of NEMO (orange), SHK (green), NEMO/SHK (red). **h** Central binding cavity occupied by SHK (carbon atoms are shown in green and oxygen atoms in red). In this zoom-in view, the surfaces of IKKβ and NEMO are shown in blue and yellow, respectively. **i** Deuterium uptake plots for the peptide located in NEMO (peptide 94–103, black) in the presence of IKKβ (orange), SHK (green), and IKKβ/SHK (red). **j** Central binding cavity occupied by SHK (carbon atoms are shown in green and oxygen atoms in red). In this zoom-in view, the surfaces of IKKβ and NEMO are shown in silver and purple, respectively

To directly investigate the SHK-binding sites within the NEMO/IKKβ complex, we conducted hydrogen–deuterium exchange mass spectrometry (HDX-MS) analyses with peptide segments covering 97.8% and 86.7% of IKKβ_701–745_ and NEMO_1–196_, respectively (Supplementary Fig. 8). According to the NEMO/IKKβ peptide complex is an asymmetrical, parallel four-helix bundle, we first compared the HDX behavior of IKKβ and NEMO alone or in the presence of the other partner. NEMO induced a significant decrease of HDX in residues 735–742 of NBD and increased the HDX in helical residues 712–717 of IKKβ relative to IKKβ alone (Fig. 5a and e). Similar results were also observed in HDX changes of NEMO induced by IKKβ, with decreased HDX in residues 94–103, and increased HDX in resides 168–187 of coiled-coil regions (Fig. 5c and f). According to these data, residues 735–742 of IKKβ and residues 94–103 of NEMO are responsible for the formation of the stabilized NEMO/IKKβ complex, which was consistent with the crystal structure of NEMO/IKKβ complex. Due to the binding of residues 735–742 and 94–103, the conformation of the peptides of IKKβ or NEMO changes, resulting in increased HDX in resides 712–717 of IKKβ or 168–187 of NEMO.

To further determine the SHK ligand-binding pockets of IKKβ and NEMO, we also evaluated its impact on the dynamics of each protein by measuring the corresponding HDX. Compared to IKKβ or NEMO alone, decreased HDX changes were also observed in NBD residues 735–742 of IKKβ and residues 94–103 of NEMO in the presence of SHK (Supplementary Fig. 7j and k). These results implied that the SHK ligand-binding pocket should be located at the bonding interface of the NEMO/IKKβ complex, thereby blocking the stable binding of NEMO/IKKβ. Previous study has shown that residues L708/V709 and L719/I723 of IKKβ were also important for NEMO/IKKβ complex formation.^52^ However, the HDX in this region was very slight, and SHK-treatment did not induce significant HDX in this region, suggesting that the action site of SHK was not located in this region.

We hypothesized that if SHK was a PPI inhibitor of NEMO/IKKβ complex formation, the HDX pattern of IKKβ or NEMO in this complex should be markedly different in the presence and absence of SHK. Accordingly, we performed HDX-MS on IKKβ in the presence of NEMO with or without SHK. Relative to NEMO only, SHK increased the HDX rate in residues 735–742 and decreased the HDX in residues 712–717 of IKKβ (Fig. 5b and e), suggesting that SHK binding resulted in a structural dissociation of IKKβ. Similar results were also observed in the corresponding region of NEMO (residues 94–103 and 168–187) in the presence of both IKKβ and SHK (Fig. 5c and f). These results suggested that SHK likely located at the bonding interface of the NEMO/IKKβ complex, and dissociated the stable binding of NEMO/IKKβ, thereby reversing HDX changes in residues 712–717, 735–742, 94–103, and 168–187.

Additionally, the deuteration of IKKβ (735–742) in the different experimental groups described above was also summarized, as shown in Fig. 5g and h. Relative to IKKβ alone (black line), we observed that SHK mildly decreased the deuteration of IKKβ (green line), while NEMO-binding caused a significant reduction in the deuteration of IKKβ (orange line). Compared to the NEMO-bound state (orange line), deuteration of IKKβ was markedly increased in the presence of SHK (red line). Consistent results were also observed in NEMO (94–103) (Fig. 5i and j), indicating that although SHK had a slight affinity for IKKβ or NEMO alone, it exhibited a strong inhibitory effect on NEMO/IKKβ complex formation. Taken together, these data support the idea that SHK binds to a vital binding domain of the NEMO/IKKΒ complex encompassing residues of both IKKβ (735–742) and NEMO (94–103), with a remarkable IC_50_ value of ~170 nM.

**Fig. 6:**
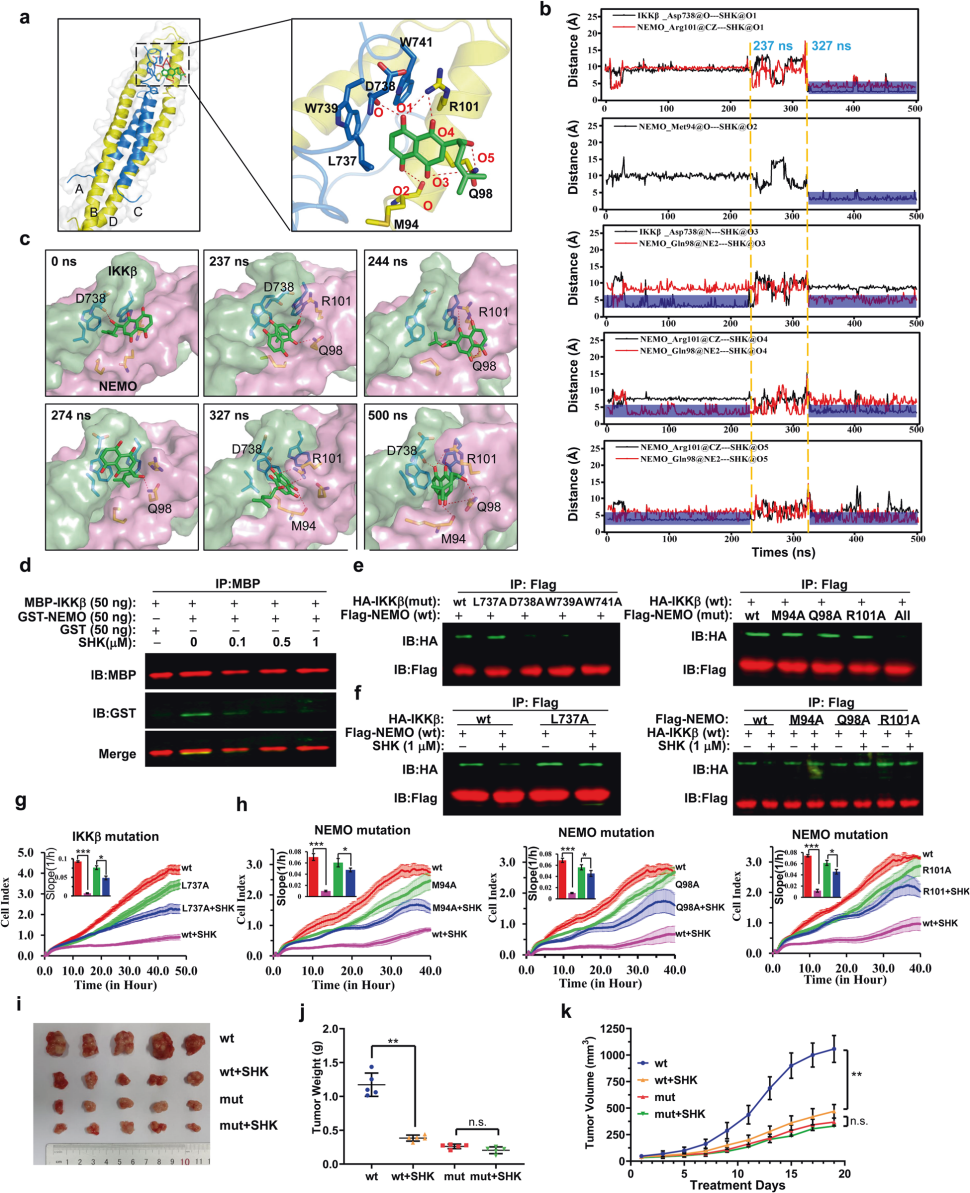
MD simulations to probe the mode of SHK binding to the NEMO/IKKβ complex. **a** MD simulations predicted the modes of SHK binding to the NEMO/IKKβ complex (left) and an enlarged view of SHK at the binding interface of the complex (right). The proteins IKKβ and NEMO are shown as blue and yellow cartoon representations, respectively. SHK and the key residues responsible for its binding are shown as stick models. The hydrogen bonds are depicted as red dotted lines. **b** The detailed changes of hydrogen bond interactions between SHK and the IKKB/NEMO complex were evaluated by the distance-based method during 500 ns of MD simulation. Blue indicates that hydrogen bonds were formed. Significant changes of hydrogen bonds occurred at 237 and 327 ns. **c** Six representative snapshots at 0, 237, 244, 274, 327, and 500 ns extracted from the MD trajectories were used to depict the dynamic binding of SHK to the NEMO/IKKΒ complex. The proteins IKKβ and NEMO are shown as light green and pink surface models, respectively. **d** Prokaryotic plasmids were used to express truncated MBP-IKKβ (701–745) and GST-NEMO (1–196) in *E. coli*. IKKβ and NEMO were pre-incubated to form a stable complex, and were then treated with SHK or left untreated. The interaction of NEMO/IKKβ was analyzed by Co-IP and western blotting using antibodies against MBP and GST. **e** HEK293T cells were co-transfected with eukaryotic plasmids expressing full-length wild-type Flag-NEMO and mutant variants of HA-IKKβ. Whole-cell extracts were collected, treated with SHK or left untreated, and analyzed by Co-IP and western blotting using antibodies against Flag and HA. **f** HEK293T cells were co-transfected with eukaryotic plasmids expressing mutant variants of full-length Flag-NEMO and wild-type HA-IKKβ. Whole-cell extracts were collected, treated with SHK or left untreated, and analyzed by Co-IP and western blotting using antibodies against Flag and HA. **g**, **h** Knockdown of IKKβ or NEMO with specific shRNA and rescue with IKKβ (wild-type) and its mutant variant (L737A) (**g**) or NEMO (wild-type) or its mutant variants (M94A, Q98A and R101A) (**h**), LoVo cells were incubated with or without SHK (1 μM). Cell index curves were generated in real time using the xCELLigence instrument. In vivo IKKβ/NEMO-dependent antitumor efficacy of SHK in a mutant *xenograft* tumor model. Tumor appearance (**i**), total tumor weights (**j**) and tumor volumes (**k**) were assessed

Since SHK could not easily form the IKKβ/SHK or NEMO/SHK binary complexes, the ternary complex (NEMO/IKKβ/SHK) might exist as an intermediate or transition state. To probe the possible binding mode of SHK with the NEMO/IKKβ complex, molecular docking followed by 500 ns of MD simulations was performed. As shown in Fig. 6a, SHK is bound to the interaction interface of the NEMO/IKKβ complex. Upon calculation of the MM/GBSA binding energy, the ligand SHK bound to the complex with a stability of −14.77 ± 3.52 kcal/mol (Supplementary Table 1). Moreover, MM/GBSA free energy decomposition analysis showed that the residues Met94, Gln98, and Arg101 of NEMO and the residues Leu737, Asp738, Trp739, and Trp741 of IKKβ significantly contributed (< −1 kcal/mol) to the binding of SHK to the NEMO/IKKβ complex (Supplementary Fig. 9). The right side of Fig. 6a shows the enlarged ternary complex diagram at 400 ns of the molecular dynamics simulation. SHK formed four hydrogen bonds with D738 (IKKβ), M94 and R101 (NEMO) at the interaction interface of the complex, and two hydrogen bonds with Q98 (NEMO) outside the interaction interface. The changes of hydrogen bond interactions between SHK and four amino acids (D738, M94, R101, and Q98) were monitored through distance measurements in real time during the MD process (Fig. 6b).

In order to further elucidate the dynamic process of SHK binding to the interaction interface of the NEMO/IKKβ complex, we selected a characteristic conformation of SHK bound to the NEMO/IKKβ complex within 500 ns of MD (Fig. 6c). During the process, the hydrogen bonds formed between SHK and Arg101 at the interface and Asn98 outside the target interface might be essential for the stable interaction between SHK and interface of the NEMO/IKKΒ complex. Taken together, the MD simulations indicated that SHK might form a relatively stable intermediate with the NEMO/IKKβ complex to interrupt the formation of the NEMO/IKKβ binary complex.

The predicted mode of PPI inhibition by SHK was partly verified by Co-IP experiments. As shown in Fig. 6d, after pre-incubation of the MBP-IKKβ (701–745) and GST-NEMO (1–196) peptides to form a stable complex, SHK still effectively reversed their association. The same results were also observed in Fig. 6f (first two lanes), whereby exogenously expressed full-length HA-IKKβ and Flag-NEMO formed stable complexes in 293T cells, and their association was also disrupted by SHK.

To validate the residues within the ligand-binding pocket of SHK, we individually mutated the residues at the binding interface of the NEMO/IKKβ complex. Firstly, we examined the effects of point mutations and found that replacement of D738, W739, W741, or M94/Q98/R101 (three points co-mutation) with alanine prevented the formation of the NEMO/IKKβ complex (Fig. 6e). By contrast, single point mutation of L737, M94, Q98, or R101 with alanine did not affect the association between IKKβ and NEMO. However, mutation of L737, M94, Q98, or R101 to alanine blocked the SHK-induced disassembly of the pre-formed NEMO/IKKΒ complex (Fig. 6f). Furthermore, we also verified the effects of these substitutions on the anti-tumor effect of SHK in real time using the xCELLigence instrument. Single point mutations have slight effects on cell growth (Fig. 6g and h, wt vs L737A, M94A, Q98A, or R101A), and the inhibitory effects of SHK were significantly weakened in the single point mutations group as compared with the wild-type group. These amino acid substitutions confirmed the importance of residues in the ligand-binding site (L737, D738, W739, W741, M94, Q98, and R101) for the disassembly of the NEMO/IKKβ complex by SHK, and further suppression of colorectal cancer progression. Besides, IKKβ/NEMO-dependent anti-tumor efficacy of SHK was also validated in an LoVo/IKKβmut (D738A/W739A/W741A, three points co-mutation) *xenograft* mouse model. As the NEMO/IKKβ complex and NF-κB pathway activation was suppressed in the LoVo/IKKβmut group, the tumor growth was slow than that of LoVo/wt group. Moreover, compared with LoVo/wt group, the inhibitory effects of SHK (5 mg/kg daily) on LoVo/IKKβ mutant group were significantly weakened (Fig. 6i-k).

To probe the important role of the OH group of SHK in the disassembly of the NEMO/IKKΒ complex, we modified the OH group at the side chain of SHK. The resulting SHK derivatives such as acetyl shikonin (ASHK), deoxyshikonin (DSHK) and methylshikonin (MSHK) are shown in Supplementary Fig. 10a. The anti-cancer effects of ASHK, DSHK or MSHK were also evaluated in colorectal cancer cells, respectively. In contrast to SHK, treatment with ASHK, DSHK, and MSHK led to only slight growth inhibition in various cancer cell lines (LoVo, RKO, SW620, HCT15, and HCT116) (Supplementary Fig. 1a and Supplementary Fig. 10b and c). Furthermore, ASHK, DSHK and MSHK lost the ability to destabilize the NEMO/IKKβ complex (Supplementary Fig. 10d and e).

## Discussion

Shikonin (SHK) is a naphthoquinone natural compound extracted from the Chinese medicinal herb *zicao*, with significant antitumor and anti-inflammatory effects.^32–34^ However, the underlying mechanism was unknown prior to this study. Here, we established a combination strategy of molecular pharmacological phenotyping, proteomics and bioinformatics analysis to identify the potential molecular target of SHK. Firstly, we used conventional molecular pharmacological methods, which revealed that SHK could significantly inhibit the expression of inflammation-related proteins, such as COX-2, iNOS, IL-6, and TNF-α (Fig. 1) to reverse the malignant phenotypes of colorectal cancer cells in vitro and in vivo, all of suggesting that anti-cancer activity of SHK is related with downregulating inflammatory pathways. Secondly, we employed TPP and SIP methods to screen the potential target proteins of SHK on a proteome-wide scale, and finally identified 22 candidate potential targets. Combined with bioinformatics analysis, the anti-inflammatory phenotypes of SHK and experimental verification, NEMO in an inflammation-related functional cluster of “Inflammatory response” was regard as the potential target. Further molecular biological results suggest SHK is a novel small-molecule PPI inhibitor of the NEMO/IKKβ complex, with an IC_50_ of ~170 nM. Therefore, we applied a practical strategy of target discovery based on molecular pharmacological phenotyping, proteomics, and bioinformatics, and further revealed the potential molecular target of SHK, which provides a useful guide for elucidation of the underlying mechanisms of pharmaceutically active natural compounds.

Diverse biochemical events depend on PPIs and the formation of protein-protein complexes. The PPI inhibitors of NEMO/IKKβ complex formation discovered to date are mainly polypeptide derivatives (NBD polypeptide, etc.).^22,53^ Although they can block the molecular interaction between IKKβ and NEMO (IC_50_ in the μM range), their poor stability, limited cell permeability and low potency greatly limit the development and clinical application of these polypeptides as drugs. Although some small-molecule inhibitors of NEMO/IKKβ complex formation were also developed using virtual screening methods on the basis of the crystal structure of this complex,^29–31^ these small-molecule PPI inhibitors demonstrate low potency against NEMO/IKKβ complex, and are active only in vitro (IC_50_ 20 µM). It is therefore urgent to develop new potent small-molecules that can enter living cells and disrupt the critical NEMO/IKKβ interaction for therapeutic implications. To our best knowledge, this is the first study to report that SHK is a potential small-molecule PPI inhibitor of NEMO-IKKβ complex formation (IC_50_ ~ 170 nM). At the same time, SHK significantly suppressed NF-κB activity with excellent anti-tumor and anti-inflammatory activities by blocking NEMO/IKKβ complex formation in vitro, in cell culture, and even in tumor *xenograft* animal models.

Previous studies revealed that the key binding regions of the NEMO/IKKβ complex were the NBD of IKKβ (L737–L742) and residues S85-Q101 of NEMO, among which W739, W741, and L742 of IKKβ are the hot spots critical for NEMO/IKKβ complex formation.^22,23,25^ The region occupied by these residues of IKKβ forms a potentially druggable binding site on NEMO that extends to IKKβ L737.^52^ Besides, NBD residues D738 is also important for binding, but not in direct contact with NEMO, instead likely acting to stabilize the active conformation of surrounding residues.^52^ In the present study, we found that SHK had a weak affinity for either IKKβ or NEMO alone, with a *K*_D_ value of approximately 10 μM, and SHK did not influence intracellular stability of IKKβ or NEMO alone. However, SHK could attenuate the association of the NEMO/IKKβ complex at nanomolar concentrations. And we further elucidated the possible mechanism through which SHK blocks the stable binding of NEMO to IKKβ. The results indicated that SHK likely binds to the bonding interface of the NEMO/IKKβ complex, and forms five intermolecular hydrogen bonds with IKKβ (D738) and NEMO (F97, Q98, and R101). These key amino acid residues of IKKβ and NEMO were confirmed to constitute the potential binding site of SHK using hydrogen-deuterium exchange mass spectrometry and amino acid point mutations. However, as for single point mutations, there is a possibility that they affect the complex structure in a way that prevents SHK binding by allosteric effects, unnecessarily SHK binding at the site of the mutation. Regardless of two possibilities, these sites were required for the formation of NEMO/IKKβ complex. Besides, there is a possibility that SHK could change the conformation of NEMO/IKKβ, resulting in the dissociation of NEMO/IKKβ. Our study thus identified the potential target of SHK, which acts as a small-molecule PPI inhibitor, providing some information for developing the PPI inhibitors of NEMO/IKKβ complex formation.

The NEMO/IKKβ complex inhibitors reported previously have a similar mode of inhibition. These inhibitors form non-covalent interactions with particular residues of NEMO, including hydrogen bonds and hydrophobic interactions. The interactions formed by inhibitors with NEMO would result in steric as well as thermodynamic barriers that hinder the binding of the incoming IKKβ subunits, thus inhibiting the formation of the active complex.^22,28^ In this study, we found that SHK probably locates in the IKKβ-binding site on the surface of NEMO (S85-Q101), and interacts with a shallow hydrophobic pocket on NEMO’s specificity surface through intermolecular hydrogen bonds and hydrophobic interactions. Therefore, It is very likely that SHK prevents the NBD of IKKβ from accessing the hydrophobic groove of NEMO, thus blocking the formation of the NEMO/IKKβ complex. Moreover, the possible ternary intermediate state of NEMO/IKKβ/SHK was studied by molecular docking and dynamics simulation. The ligand-binding pocket of SHK predicted from our simulation was further validated by mutations and Co-IP assays. Replacement of L737, M94, Q98 or R101 with alanine did not affect the association between IKKβ and NEMO, while effectively blocking the SHK-induced disassembly of the IKKβ /NEMO complex (Fig. 6e). These results indicate that SHK not only blocked the complex formation, but also effectively dissociated the pre-formed complex. NEMO and IKKβ protein form large amounts of stable complexes under pro-inflammatory stimulation, which can promote the occurrence and progression of various tumors. However, due to its inhibition mode based on the dissociation of the NEMO/IKKβ complex, SHK might still exhibit significant anti-cancer activity, even though NEMO and IKKβ have formed a stable complex in cancer cells after inflammatory stimulation. Accordingly, this inhibition mode makes SHK a potential inhibitor of NEMO/IKKβ complex formation to block NF-κB activation in vitro and in vivo, so that it could effectively treat colorectal cancer.

In summary, using a combination strategy of molecular pharmacological phenotyping, proteomics and bioinformatics analysis, we discover that SHK is a small-molecule PPI inhibitor of the NEMO/IKKβ interaction, with an IC_50_ of approximately 170 nM. And the potential mechanism through which SHK inhibits NEMO/IKKβ complex formation uncovered in this study, will usefully lead to effective treatments for inflammatory diseases and cancer.

## Materials and methods

### Thermal proteome profiling (TPP) and solvent-induced protein precipitation (SIP) assay

For the TPP assay, LoVo cells were harvested and lysed using PBS containing 1% EDTA-free cocktail at pH 7.4. The cell suspensions were frozen in liquid nitrogen for three times, and the supernatant was divided into two aliquots, and treated with 100 μM SHK and DMSO alone as vehicle control. After the incubation for 25 min at room temperature, the two mixtures were divided into five aliquots, respectively. The five replications were heated individually at 52 °C for 3 min in a PCR machine. The heated lysates were centrifuged to separate soluble proteins from precipitated proteins. The supernatants were collected for quantitative proteomics analysis. The protein concentration was determined using a BCA protein assay kit (Thermo Fisher Scientific, San Jose, CA, USA).

For the SIP assay, LoVo cells were lysed and incubated with DMSO or SHK as described in the TPP method above. The five replications were treated with an organic solvent mixture comprising acetone: ethanol: acetic acid at a ratio of 50: 50: 0.1 to reach a final solvent concentration of 16%. Subsequently, the mixtures were equilibrated at 800 rpm for 20 min at 37 °C. The supernatants were used for quantitative proteomics analysis.

### Co-immunoprecipitation (co-IP) assay

HEK293T cells were co-transfected with plasmids encoding full-length HA-IKKβ and Flag-NEMO, and then treated with SHK for 48 h. The pre-cleared cell lysates were incubated with an anti-HA antibody on a rotating platform at 4 °C for 2 h, and then incubated for another 6 h with protein A/G-sepharose beads. The sepharose beads were washed three times with binding buffer, mixed with gel loading buffer and boiled for 5 min. The samples were centrifuged and the supernatants were used for western blot analysis. A specific antibody against the Flag-tag was applied for the detection of proteins.

### Western blot analysis

Protein lysates were separated by electrophoresis in a 10% acrylamide sodium dodecyl sulfate-polyacrylamide minigel (SDS-PAGE), electrophoretically transferred to a PVDF membrane, and immunoblotted with specific antibodies. The protein bands were detected by enhanced chemiluminescence. The protein concentrations were determined using a BCA protein assay kit (Beyotime Biotechnology, China). The experiments were carried out at least three times.

### In vitro transfection assay

The IKKβ and NEMO expression vectors were constructed by subcloning the full-length cDNA to pENTER-4T1 expression vector (Flag- or HA-tagged) developed by ViGene Biosciences, as described previously.^25^ The 293T cells (1 × 10^6^) were plated into 60 mm dishes for 1 day, and then transfected with the relevant plasmids encoding HA-IKKβ and Flag-NEMO using Lipofectamine 2000 transfection reagent (Invitrogen). After transfection, the cells were cultured for another 24 h. Subsequently, the transfected cells were trypsinized and seeded into 60 mm dishes, followed by culturing with fresh medium containing the indicated doses of SHK for 48 h, and then collected for the Co-IP assay.

### Computational modeling

The Surflex-Dock module of Sybyl-X2.1 software (Tripos Associates Inc., S.H.R.; St. Louis, MO 631444, USA) was used for molecular docking to predict the binding mode of SHK with the NEMO/IKKβ complex. The docking parameters were set to default. The top-ranking docking pose was further assessed by MD simulations to determine the minimum energy conformation of the NEMO/IKKβ/SHK ternary complex. MD simulations were performed using AMBER12 with the AMBER FF99SB force field for proteins and the GAFF force field for the ligand. The partial charge for the ligand SHK was developed using RESP charge fitting based on HF/6–31* electrostatic potential. Then, the system was immersed into a cubic TIP3P water box extending 12 Å from any solute atom, and KCl was added at a final concentration of 0.1 M to neutralize the system. The system was minimized using the conjugate gradient and steepest descent methods as described in previous papers.^54,55^ Subsequently, the system was gradually heated from 0 to 310 K over 60 ps. Finally, a 500 ns MD simulation with a 2 fs time step was performed in triplicate for the system at a constant temperature of 310 K. Throughout all simulations the SHAKE procedure was employed to constrain all bonds involving at least one hydrogen atom, and the particle mesh Ewald (PME) method was adopted to deal with long-range electrostatic interactions. Analysis was performed on frames spaced by 0.1 ns using the cpptraj module and Molecular Mechanics/Generalized Born Surface Area (MM/GBSA) pairwise energies were calculated for the interaction between SHK and the NEMO/IKKβ complex.

### Surface plasmon resonance (SPR) assay

#### For MBP-IKKβ

MBP-IKKβ was immobilized on the surface of the CM5 chip using the amine-coupling approach at a flow rate of 10 μL/min in 10 mM sodium acetate buffer (pH 5.0). The sensor surface was activated with a 7 min injection of a mixture comprising 50 mM N-hydroxysuccinimide (NHS) and 200 mM 1-ethyl-3-(3- dimethylaminopropyl) carbodiimide (EDC). Then, 33 μg/mL of IKKβ was injected to reach the target level of 500 RU and the surface was blocked with 1 M ethanolamine, pH 8.5. A series of concentrations of NEMO or SHK were injected into the flow system and analyzed for 90 or 120 s, followed by dissociation for 90 or 600 s. All binding analyses was performed in phosphate-buffered saline (PBS, pH 7.4) with 0.05% (v/v) Tween-20 at 25 °C. Prior to analysis, double reference subtractions were made to eliminate bulk refractive index changes, injection noise, and data drift. The binding affinity was determined by fitting to a Langmuir 1:1 binding model in the Biacore Evaluation software (GE Healthcare).

#### For GST-NEMO

According to the instructions from GE, an anti-GST antibody was immobilized onto the CM5 chip surface. The sensor surface was activated with NHS and EDC, after which 15 μg/mL of anti-GST antibody was injected for 420 s and the surface was blocked with 1 M ethanolamine, pH 8.5. After immobilization, recombinant GST protein was injected into the chip and regenerated with 10 mM glycine-HCl (pH 2.1) three times to block the high-affinity sites. For each analyte, 50 μg/mL of GST-G was captured for 60 s and 10 mM glycine-HCl (pH 2.1) was used to regenerate the surface. MBP-B was analyzed with 120 s injection and 120 s dissociation. A series of SHK concentrations was analyzed with 90 s injection and 90 s dissociation.

For the competition assay, we tried two methods with MBP-IKKβ and GST-NEMO immobilized separately. Mixtures including series concentrations of SHK and 1 μM GST-NEMO were tested on MBP-IKKβ-immobilized surface; while other mixtures including series concentrations of SHK and 0.5 μM MBP-IKKβ were tested on GST-NEMO-captured surface. For the MBP-IKKβ-immobilized surface, 10 mM Glycine-HCl pH 2.1 was injected for 30 s to remove the rest bound NEMO after dissociation. For the GST-NEMO-captured surface, a regeneration with 10 mM Glycine-HCl pH 2.1 was carried out for 30 s to remove both the bound IKKβ and the captured GST-NEMO after each dissociation. Fresh GST-NEMO would be captured again for the analysis of the following sample. Based on the affinity binding assay described above, various concentrations of SHK were added into 1 μM GST-NEMO (or 0.5 μM MBP-IKKβ) and incubated at room temperature for 30 min. Then, samples were injected to determine the binding to MBP-IKKβ (or GST-NEMO) on the chip surface. By recording the binding responses at 120 s, the inhibition effects of SHK were calculated to determine the IC_50_.

### Hydrogen/deuterium exchange

Prior to HDX labeling, proteins were diluted to 180 μM in phosphate-buffered saline (pH 7.4), and incubated alone or with SHK (3 μM) for 30 min. To initiate deuterium labeling, 5 μl of each 180 μM protein solution was diluted with 45 μl of labeling buffer (20 mM Tris, 500 mM (NH)_2_SO_4_, 99% D_2_O, pH 7.0) at 25 °C. To terminate deuterium labeling, the labeling reaction was quenched by adding 50 μl of ice-cold quenching buffer (4 M guanidine hydrochloride, 200 mM citric acid and 500 mM TECP in water at pH 1.8, 100% H_2_O), after which the samples were immediately put on ice. Then, 5 μl of a 1 μM pepsin solution was added for digestion. At 3 min, the sample was placed into a Thermo-Dionex Ultimate 3000 HPLC system autosampler for injection.

For LC-MS/MS analysis, the peptides were separated at a flow rate of 115 µl/min by a 20 min gradient elution on a Thermo-Dionex Ultimate 3000 HPLC system, which was directly interfaced with a Thermo Scientific Q Exactive mass spectrometer. Peptides were separated on a reversed-phase column (Acquity UPLC BEH C18 column 1.7 µm, 2.1 × 50 mm, Waters, UK). Mobile phase A consisted of 1% formic acid in water, and mobile phase B consisted of 100% acetonitrile and 1% formic acid. The Q Exactive mass spectrometer was operated in the data-dependent acquisition mode using Xcalibur 2.0.0.0 software with a single full-scan mass spectrum in the orbitrap (350–2000 *m/z*, 70,000 resolution). The mass spectrometer was operated using a source temperature of 250 °C and a spray voltage of 3.0 kV. Peptic peptides were identified using Proteome Discoverer software (Version PD1.4, Thermo-Fisher Scientific, USA). The search criteria were as follows: full tryptic specificity was required; two missed cleavages were allowed; precursor ion mass tolerances were set at 20 ppm for all MS acquired in an orbitrap mass analyzer; and the fragment ion mass tolerance was set at 0.02 Da for all acquired MS2 spectra. The false discovery rate (FDR) of peptides was calculated using Percolator provided by PD. When the q value was smaller than 1%, the peptide spectrum match (PSM) was considered to be correct. FDR was determined based on PSMs searched against the reverse, decoy database. Peptides only assigned to a given protein group were considered unique. The false discovery rate (FDR) was also set to 0.01 for protein identification. The deuterium exchange levels were determined by subtracting the centroid mass of the non-deuterated peptide from the centroid mass of the deuterated peptide using HDExaminer (Version PD1.4, Thermo-Fisher Scientific, USA).

## Supplementary information

Supplementary Infornation

## Data Availability

All data and associated protocols are included in the manuscript and available from the corresponding author upon reasonable request.
