# Correction: Characterization of a small-molecule inhibitor targeting NEMO/IKKβ to suppress colorectal cancer growth

**DOI:** 10.1038/s41392-023-01618-x

**Published:** 2023-09-04

**Authors:** Zhenlong Yu, Jian Gao, Xiaolei Zhang, Yulin Peng, Wenlong Wei, Jianrong Xu, Zhenwei Li, Chao Wang, Meirong Zhou, Xiangge Tian, Lei Feng, Xiaokui Huo, Min Liu, Mingliang Ye, De-an Guo, Xiaochi Ma

**Affiliations:** 1 Pharmaceutical Research Center, Second Affiliated Hospital, Dalian Medical University, Dalian, 116000 China; 2 College of Pharmacy, College (Institute) of Integrative Medicine, Dalian Medical University, Dalian, 116044 China; 3 Jiangsu Key Laboratory of New Drug Research and Clinical Pharmacy, Xuzhou Medical University, Xuzhou, 221004 China; 4 CAS Key Laboratory of Separation Sciences for Analytical Chemistry, National Chromatographic R&A Center, Dalian Institute of Chemical Physics, Chinese Academy of Sciences, Dalian, 116023 China; 5 Shanghai Research Center for Modernization of Traditional Chinese Medicine, National Engineering Research Center for TCM Standardization Technology, Shanghai Institute of Materia Medica, Chinese Academy of Sciences, Shanghai, 201203 China; 6 Academy of Integrative Medicine, Shanghai University of Traditional Chinese Medicine, 1200 Cailun Road, Shanghai, 201203 China; 7 Neurology Department, Dalian University Affiliated Xinhua Hospital, Dalian, 116021 China

**Keywords:** Drug development, Target identification

Correction to: *Signal Transduction and Targeted Therapy* 10.1038/s41392-022-00888-1, published online 09 March 2022

In the process of collating the raw data, the authors noticed an inadvertent mistake occurred in Fig. 3b that needs to be corrected after online publication of the article.

In Fig. 3b, as a result of an error in the graphics panel arrangement process, the band of NEMO was repeatedly inserted as β-actin by mistake. The correct band is shown as below and in the updated Fig. 3b. The correction did not affect any of our results or discussion as present in the original publication. We regret any inconvenience this has caused.
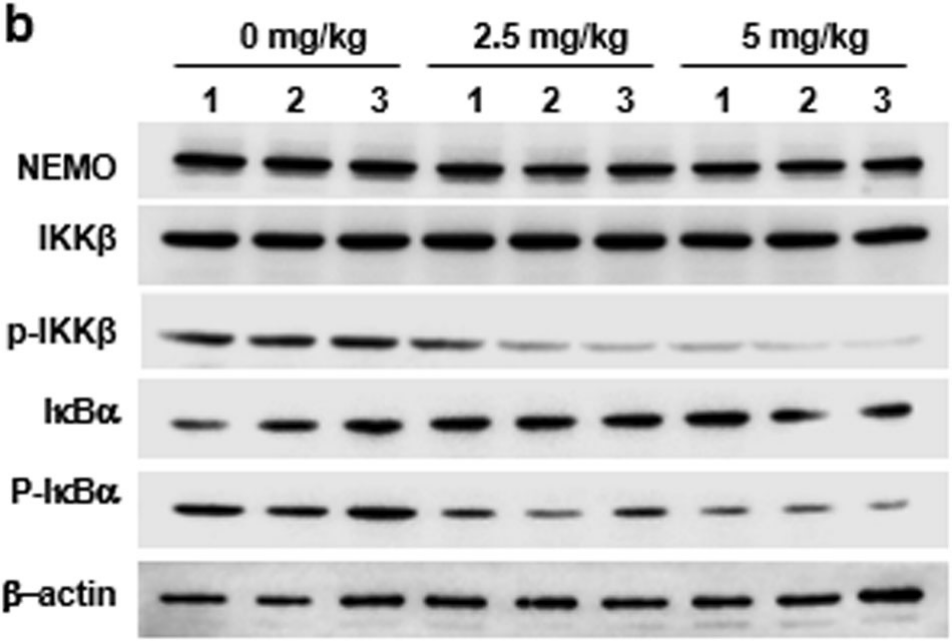


The original article has been corrected.

