# Supplementary material for: Characterization of a small-molecule inhibitor targeting NEMO/IKKβ to suppress colorectal cancer growth
Source: Signal Transduct Target Ther. 2022 Mar 9;7:71. doi: 10.1038/s41392-022-00888-1 (PMC8904520; doi:10.1038/s41392-022-00888-1)
Supplement: Supplementary file 1 — Supplementary Infornation [file 41392_2022_888_MOESM1_ESM.docx]

Supplementary Materials for

**Characterization of a small-molecule inhibitor targeting NEMO/IKKβ to suppress colorectal cancer growth**

Zhenlong Yu^1,2^, Jian Gao^3^, Xiaolei Zhang^4^, Yulin Peng^1,2^, Wenlong Wei^5^, Jianrong Xu^6^, Zhenwei Li^5^, Chao Wang^2^, Meirong Zhou^2^, Xiangge Tian^1^, Lei Feng^1^, Xiaokui Huo^1^, Min Liu^2,7^, Mingliang Ye^4^, De-an Guo^5^, Xiaochi Ma^1,2^

^1^ Pharmaceutical Research Center, Second Affiliated Hospital, Dalian Medical University, Dalian 116000, China

^2^ College of Pharmacy, College (Institute) of Integrative Medicine, Dalian Medical University, Dalian 116044, China;

^3^ Jiangsu Key Laboratory of New Drug Research and Clinical Pharmacy, Xuzhou Medical University, Xuzhou 221004, China;

^4^ CAS Key Laboratory of Separation Sciences for Analytical Chemistry, National Chromatographic R&A Center, Dalian Institute of Chemical Physics, Chinese Academy of Sciences, Dalian 116023, China;

^5^ Shanghai Research Center for Modernization of Traditional Chinese Medicine, National Engineering Research Center for TCM Standardization Technology, Shanghai Institute of Materia Medica, Chinese Academy of Sciences, Shanghai 201203, China;

^6^ Academy of Integrative Medicine, Shanghai University of Traditional Chinese Medicine, 1200 Cailun Road, Shanghai, 201203, China;

^7^ Neurology Department, Dalian University Affiliated Xinhua Hospital, Dalian 116021, China.

**Correspondence:** Mingliang Ye ([mingliang@dicp.ac.cn](mailto:mingliang@dicp.ac.cn), Tel: +86 411-84379610; Fax: +86 411-84379610), De-an Guo ([daguo@simm.ac.cn](mailto:daguo@simm.ac.cn), Tel: +86 021-50271516; Fax: +86 021-50271516), Xiaochi Ma ([maxc1978@163.com](mailto:maxc1978@163.com), Tel: +86 411-86110419; Fax: +86 411-8611 0419).

**This PDF file includes:**

Materials and Methods

Figures. S1 to S10

Tables S1

**Materials and Methods**

**Reagents and antibodies**

Shikonin (SHK; JOT-11729, ≥98%) was purchased from Chengdu Pufei De Biotech Co., Ltd (China), and dissolved in DMSO to produce a 5 mM stock solution. The primary antibodies against iNOS, COX-2, IL-6, TNF-α, IKKβ, p-IKKα/β, IκB-α, p-IκB-α, NF-κB p65, cleaved-PARP, cleaved-caspase 3/9, and all the secondary antibodies were obtained from Cell Signaling Technology (USA). The primary antibodies against CDK4, cyclin D1, cyclin E1, HA, Flag, MBP, GST, Nrf2, HO-1, NEMO, Lamin B1, β-actin and cytochrome c were obtained from Proteintech Group (USA). The primary antibodies against MMP2/9, TIMP2 were obtained from Abcam. Dulbecco’s Modified Eagle’s Medium (DMEM), RPMI 1640, fetal bovine serum (FBS), and trypsin were obtained from HyClone Laboratories (USA). All other chemicals were purchased from Sigma Chemical Co. (St. Louis, MO) unless specified otherwise.

**Cell lines and cell culture**

Human colon cell lines LoVo, RKO, SW620, HCT116, HCT15 and CCD841 CoN were obtained from the American Type Culture Collection (ATCC Manassas, VA, USA) or the Procell Life Science & Technology Co., Ltd (Wuhan, China), and preserved in our laboratory. These cells were cultured in DMEM or 1640 medium supplemented with 10% FBS, 100 μg/ml penicillin and 100 μg/ml streptomycin at 37°C in a humidified atmosphere comprising 5% CO_2_. The authenticity of all cell lines was verified through genomic short tandem repeat profiling, and the cell lines were confirmed to be free of mycoplasma using the Mycoplasma Detection Kit-Quick Test (Biotool, Houston, TX, USA).

**Cell viability assay**

Cell viability was determined using the CCK-8 assay. Briefly, 5×10³ cells were seeded into 96-well culture plates, allowed to adhere for overnight, and then changed to fresh medium containing various concentrations of SHK (0, 0.5, 1, 2 and 5 µM) dissolved in DMSO (final concentration, 0.1%). After incubation for 48 h, the CCK-8 reagent was added, and the absorbance was measured at 450 nm using an *EnSpire*® Multimode Plate Reader (Perkin Elmer, USA). The cell viability in the vehicle control groups was defined as 100%. Each assay was carried out in triplicate.

**Colony formation assay**

Detached cells (1 × 10^3^ per well) were seeded into six-well plates containing 2 mL of growth medium with 10% FBS and cultured for 24 h. Then, the medium was removed and the cells were treated with various concentrations of SHK. After 24 h, the cells were washed with PBS and supplemented with fresh growth medium. The cells were routinely incubated for about two weeks until colonies were large enough to be visualized. Then, the colonies were stained with 0.1% crystal violet and counted.

**Nitric Oxide (NO) Production Assay**

After overnight culture in a 96-well plate (1 × 10^4^ cells in 100 μL medium per well), the cells were pre-treated with SHK for 1 hour and lipopolysaccharide for an additional 24 hours. The culture supernatant from each well was collected at the end of the experiment and used to measure NO production. The NO production was determined using a commercial NO assay kit on the basis of the Griess reaction (S0021, Beyotime Institute of Biotechnology, Shanghai, China) according to the manufacturer’s instructions.

***In vitro* migration assay**

The scratch assay (wound healing assay) was performed to assess the cell migration ability. The cells were grown to full confluence in six-well plates, after which the resulting monolayers were wounded with a sterile 200 μl pipette tip and then washed with PBS after 6 h of starvation. The cells were changed to fresh medium with 5% FBS containing the indicated doses of SHK. After 48 h, medium was replaced with PBS, the wound gap was observed, and cells were photographed using a Leica DM14000B microscope fitted with a digital camera and the distance of the wound gap was measured.

**Flow cytometry analysis**

To determine the cell cycle distribution and the proportion of apoptotic cells, we performed flow cytometry analysis using a FACS Accuri C6 flow cytometer (BD, CA, USA). For cell-cycle analysis, cells were treated with SHK (0, 0.5, and 1 µM) for 48h, then trypsinized and washed with PBS, resuspended in chilled 70% methanol, and kept overnight at 4°C. The fixed cells were then collected, stained with propidium iodide (PI) staining buffer (0.2% Triton X-100, 100 μg/mL DNase-free RNaseA, and 50 μg/mL PI in PBS) and incubated at 37°C ­for 30 minutes. For the apoptosis examination, the cells were washed with PBS, and stained using the Annexin V-FITC Apoptosis Detection Kit in the dark at room temperature for 15 min. The cell cycle distribution and the fraction of apoptotic cells were determined using a FACS analysis system. Each experiment was performed in triplicate.

**Immunofluorescence staining and confocal microscopy**

LoVo cells grown on chamber slides in medium with the indicated SHK concentrations were fixed with 4% paraformaldehyde and permeabilized with 0.2% TritonX-100. The samples were probed with specific antibodies against Cytochrome c (cyt c), p50 or p65 (Santa Cruz), followed by secondary antibodies conjugated with fluorescein isothiocyanate or rhodamine. Subsequently, the cell nuclei were counterstained with DAPI. The samples were examined under a Leica DM14000B confocal microscope.

**Streptavidin-agarose pull-down assay for the detection of DNA-protein binding**

The binding of NF-κB p65/p50 to COX-2 promoter probes was determined using a streptavidin-agarose pull-down assay. A 478-bp biotin-labeled double stranded probe corresponding to the COX-2 promoter sequence (0 to -478) was synthesized. Briefly, 400 µl mixtures containing a nuclear protein extract (400 μg), biotinylated DNA probe (4 μg), streptavidin-conjugated agarose beads (40 μl) and PBSi (PBS buffer with 1 mM EDTA, 1 mM DTT and protease inhibitor cocktail complete) were incubated at room temperature for 5 h on a rotating rack. After washing with PBSi buffer, the beads were resuspended in SDS-PAGE loading buffer and boiled at 100 °C. The supernatant was analyzed by western blotting.

**Dual-luciferase reporter assay**

LoVo cells in a 6-well plate were transfected with the pNFκB-luc plasmid (Beyotime), which contains the luciferase coding region downstream of an NFκB-responsive promoter, using Lipofectamine 2000 Reagent (Invitrogen, Grand Island, NY, USA). After transfection, the cells were treated with SHK (0.5 and 1µM) for 48 h. The dual-luciferase reporter assay system (Promega, Madison, WI) was used to measure the changes of luciferase activity in the cell lysates. The firefly luciferase activities were normalized to the corresponding *Renilla* luciferase activities. All values are shown as means ± SD of triplicate experiments.

**Protein Expression and Purification**

The GST-NEMO (1-196) expression vector was derived from pGEX-6p-1 (GE Healthcare), and the MBP-IKKβ expression vector was derived from pMAL-c5X. The proteins were produced in *E. coli* BL21(DE3) cells grown at 20 ℃ and induced with 1 mM IPTG. Cells were re-suspended in lysis buffer (25mM Tris-HCl pH 8.0, 500mM NaCl, 5% Glycerol, 1mM TCEP-HCl, 1×PIC,) and subjected to homogenization using a high-pressure cell disruptor before centrifuging at 15000g and 4℃ for 1h. The supernatant (cleared lysate) was incubated with GSH-Sepharose beads or amylose agarose beads (NEB) (column volume (CV): 2.5 mL) under shaking at 4℃ overnight. The beads were loaded onto a gravity column and the flow-through was collected. The column was washed with 30CV (75 mL) (10CV/drip-wash, 3 times washes) of wash buffer (lysis buffer without PIC), and eluted with 10 CV (25 mL) of elution buffer (lysis buffer with 20mM D-(+)-maltose, without PIC).

**FRET Activity Assays**

A FRET assay was used to detect the effects of SHK on NEMO/IKKβ binding. 10 nM GST-NEMO_1–196_ was bound to 200 nM biotinylated IKKβ peptide in binding buffer (50 Mm Tris-HCl, 150 mM NaCl, and 1x cocktail, pH 7.4), and increasing amounts of SHK were titrated into the mixture, incubated for 30 min at room temperature. The detection agent (50 nM streptavidin-conjugated APC and 10 nM LANCE^TM^ Europium-W1024 labeled anti-GST, Perkin Elmer) was incubated for 30 min at room temperature, and the FRET signal was read on a multifunctional microplate reader (BioTek Synergy NEO).

**Animal experiments**

Female athymic nude mice aged 5 weeks were purchased from Beijing Vital River Laboratory Animal Technology Co., Ltd., and kept in the SPF Laboratory Animal Center of Dalian Medical University (Dalian, China). All animals were given free access to sterilized food and water and were habituated for 7 days before the experiments. All procedures were carried out in strict accordance with the recommendations established by the Animal Care and Ethics Committee of Dalian Medical University as well as the guidelines promulgated in the U.S. National Institutes of Health Guide for the Care and Use of Laboratory Animals. LoVo cells (LoVo/wt and LoVo/ mut, 5×10^6^ in 100 μL PBS) were injected subcutaneously near the axillary fossa of each nude mouse. Two weeks later, when the formed tumor reached 3-4mm, the animals were divided randomly into three groups with 5 mice in each group. Group A was treated with PBS as negative control, group B with 2.5 mg/kg SHK, and group C with 5 mg/kg SHK by intraperitoneal injection once every two days. The tumors were measured with a caliper once every two days, and the tumor volume was calculated using the formula V = 1/2 (width^2^ × length). The body weight of the animals was also recorded. After treatment with SHK for 15 days, all experimental mice were sacrificed and the tumors from each mouse were excised and their weight was measured. To determine the expression of the indicated proteins, the tumor tissues were fixed with 10% neutral formalin and embedded in paraffin. Then, 4 μm sections were stained with hematoxylin and eosin, in combination with specific antibodies (iNOS (1:100), COX-2 (1:50), IL-6 (1:100)), and TNFα (1:200)). The stained samples were examined under a Leica DM 4000B fluorescence microscope equipped with a digital camera. The other parts of the tumors were used to prepare tumor tissue lysates for western blot analysis.

**Accession codes.** Protein Data Bank (PDB): Structures have been deposited under accession codes 3BRV for NEMO-IKKβ complex.

**Statistical analysis**

All experiments were repeated at least three times. Data are presented as means ± standard deviation (SD). Analysis of variance and Student’s *t*-test were used to compare the values of the test and control samples. Differences with P < 0.05 were considered statistically significant. SPSS 17.0 (IBM Corp., USA) was used for all statistical analyses.

**
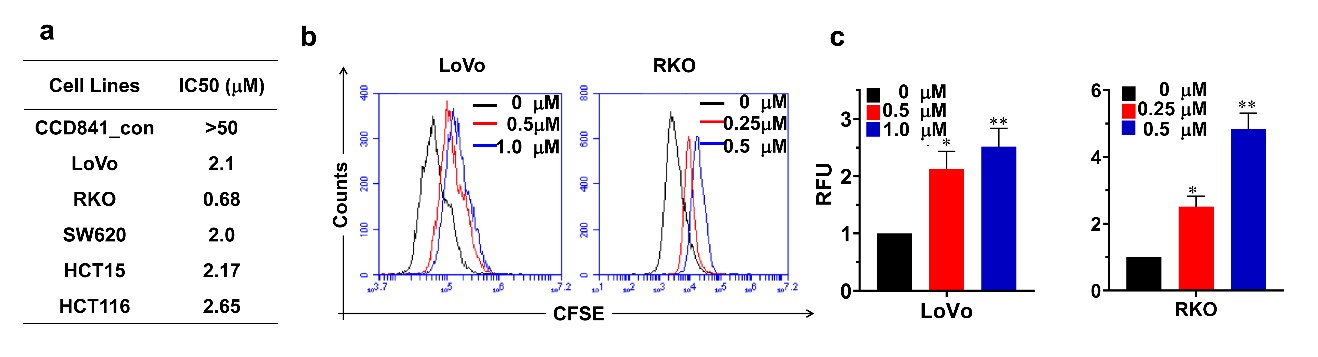
**

**Figure. S1.** **Effect of SHK on the proliferation of colon cancer cells. (a)** The IC_50_ values of SHK for cell viability inhibition in human colorectal cancer cells (LoVo, RKO, HCT-15, HCT-116 and SW620) and human normal colonic epithelial cells (CCD841 CoN) were determined. **(b)** LoVo and RKO cells were stained with CFSE and cultured with SHK for 48h. Cell proliferation was determined using a BD Accuri C6 Flow Cytometer. **(c)** The relative fluorescence units (RFU) of CFSE staining were calculated. The data are presented as the means ± SD of at least three separate experiments. (*p< 0.05, **p< 0.01, SHK treatment group vs. vehicle control group)

**
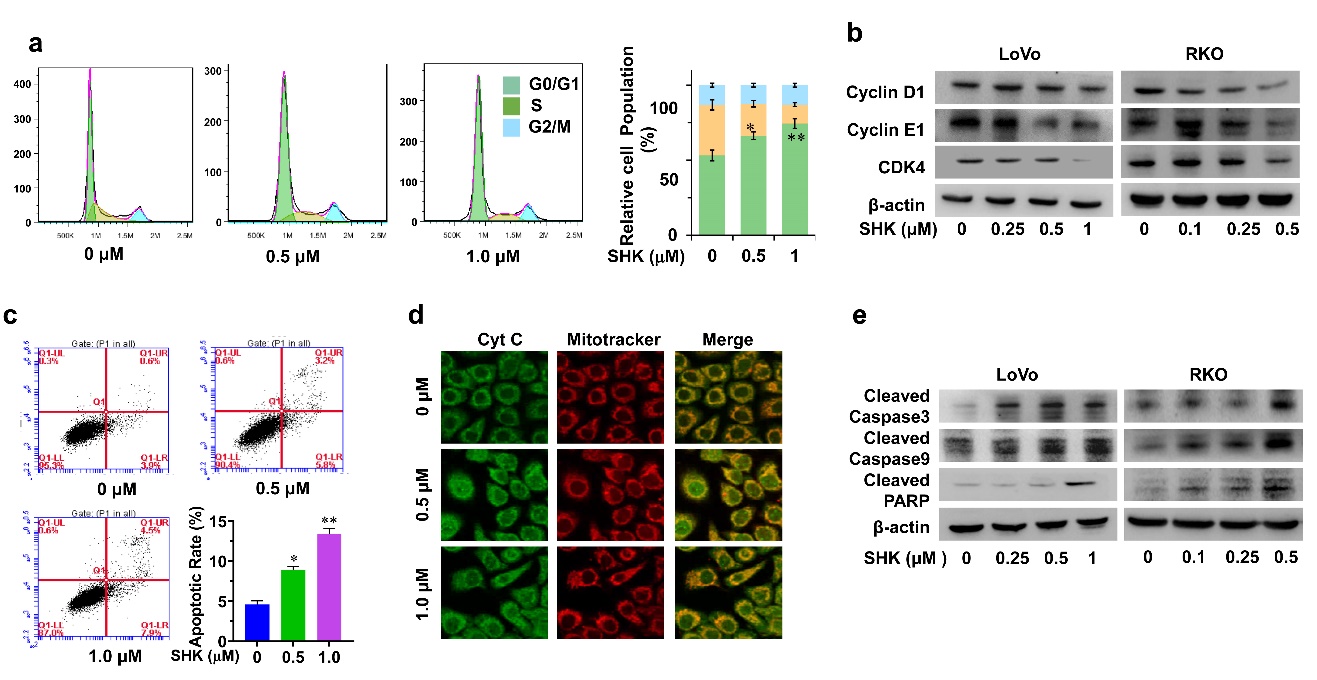
**

**Figure. S2. Effects of SHK on cell cycle and apoptosis in colorectal cancer cells.** LoVo and RKO cells were treated with SHK at the indicated doses for the indicated time. **(a)** The cell cycle analysis of LoVo cells was performed using a BD Accuri C6 Flow Cytometer. The percentage of cells at each phase of the cell cycle was quantified. **(b)** The expression of the CDK4, cyclin D1, and cyclin E1 proteins was analyzed by western blotting. **(c)** Apoptosis was determined by FACS analysis, and the percentage of apoptotic cells was calculated. **(d)** The release of cytochrome c from mitochondria to the cytoplasm in apoptotic LoVo cells was observed by immunofluorescence imaging. **(e)** The protein levels of cleaved caspase-3/9 and cleaved PARP were analyzed by western blotting. Data were presented as the means ± SD of at least three independent experiments. (*p< 0.05, **p< 0.01, SHK treatment vs. vehicle control groups).

**
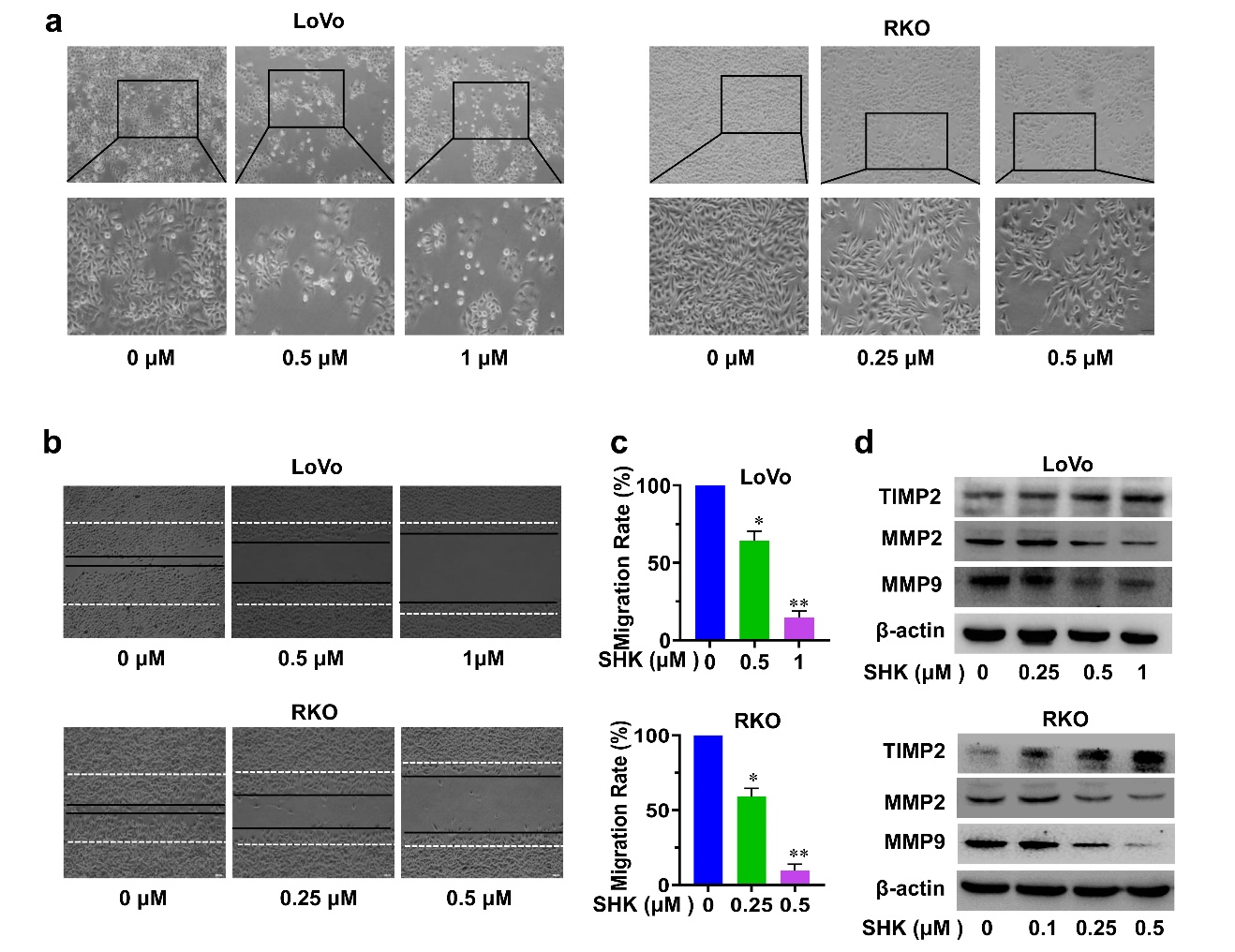
**

**Figure. S3. Effects of SHK on the morphology and migration ability of colorectal cancer cells. (a)** The changes of cell morphology and spreading in LoVo and RKO cells cultured with the indicated concentrations of SHK for 48h were observed, and photographed using a microscope fitted with a digital camera (magnification, 200× (upper), 400× (bottom)). **(b)** Cell migration was analyzed using a scratch assay. After treatment with SHK for 48h, the wound gap was observed and photographed (magnification, 100×). **(c)**The percentage of migrating cells was calculated relative to the original gap. Data were presented as the means ± SD of at least three independent experiments. (*p< 0.05, **p< 0.01, SHK treatment group vs. vehicle control group). **(d)** The expression of TIMP-2 and MMP-2/9 proteins was analyzed by western blotting.

**
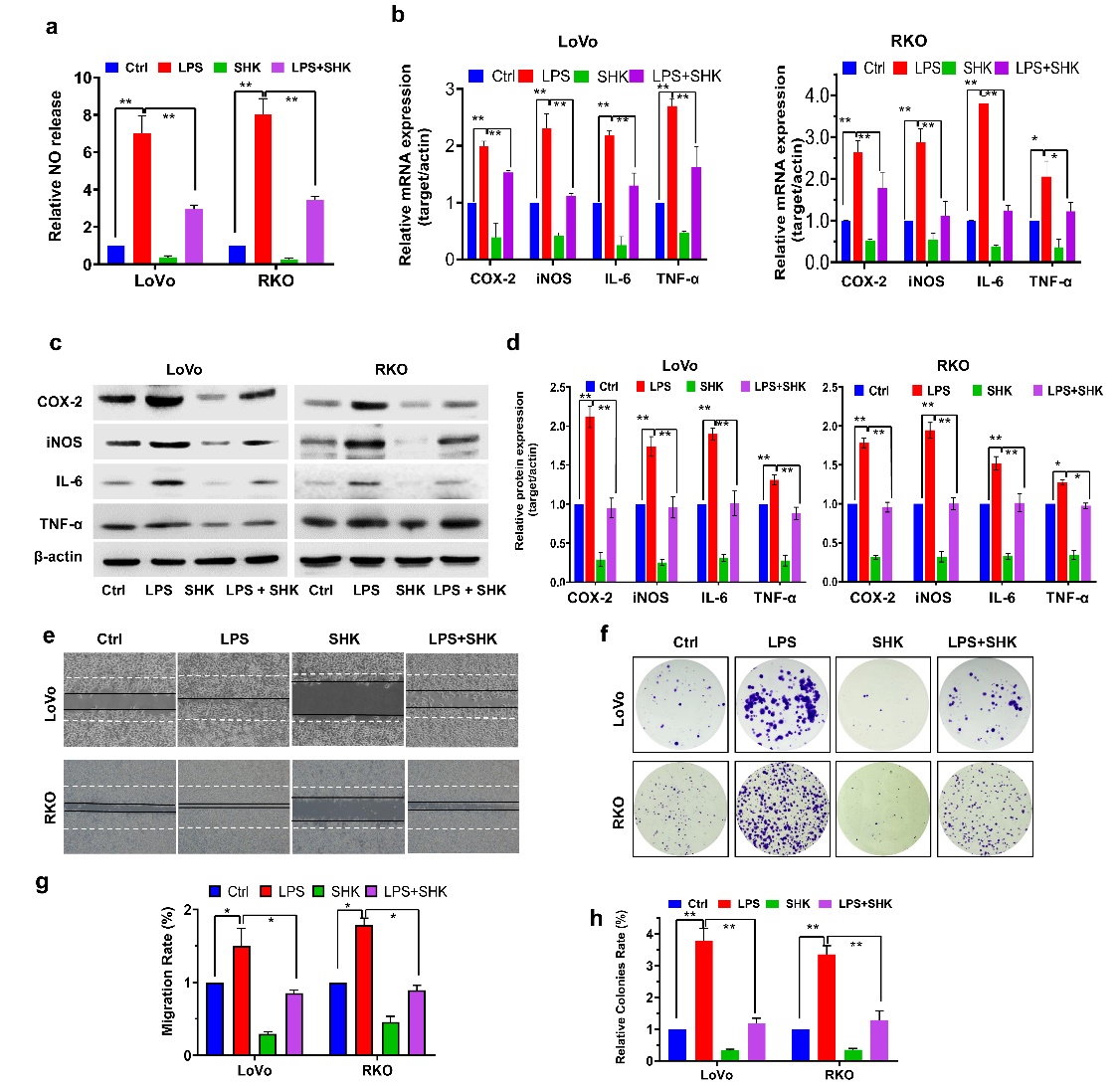
**

**Figure. S4.** **Effects of SHK on pro-inflammatory signaling in colorectal cancer cells induced by LPS.** LoVo and RKO cells were pre-treated with SHK (1 μM) for 1 hour and treated with LPS for an additional 24 hours. The NO content was determined using Griess reagent **(a)**, the expression of pro-inflammatory cytokines was measured by qPCR assay **(b)**, and the expression of inflammatory markers (COX-2, iNOS, IL-6, and TNF-α) was analyzed by western blotting **(c)**. A quantitative analysis of these proteins was also performed **(d)**. Cell migration was analyzed using a scratch assay **(e)** (magnification, 100×), and the migration rate was calculated **(g)**. The SHK-induced decrease of colony formation was analyzed with or without LPS-treatment **(f)**, and the colony formation numbers were calculated **(h)**. The data are presented as the means ± SD of at least three separate experiments. (*p< 0.05, **p< 0.01, SHK treatment group vs. vehicle control group).

**
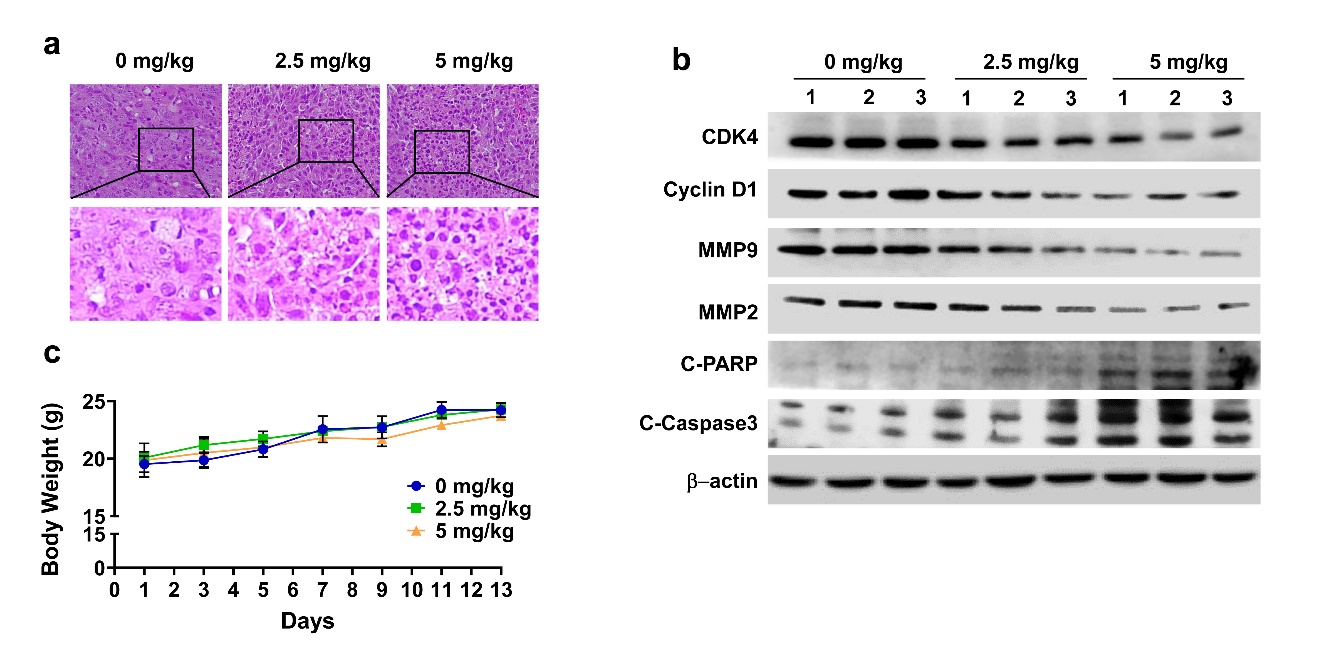
**

**Figure. S5. *In vivo* antitumor efficacy of SHK in a LoVo xenograft tumor model.** **(a)** H&E staining. **(b)** The protein levels of CDK4, CyclinD1, MMP2/9, cleaved PARP and cleaved caspase3 in tumor tissues were analyzed by western blotting. **(c)** Body weight of the mice.

**
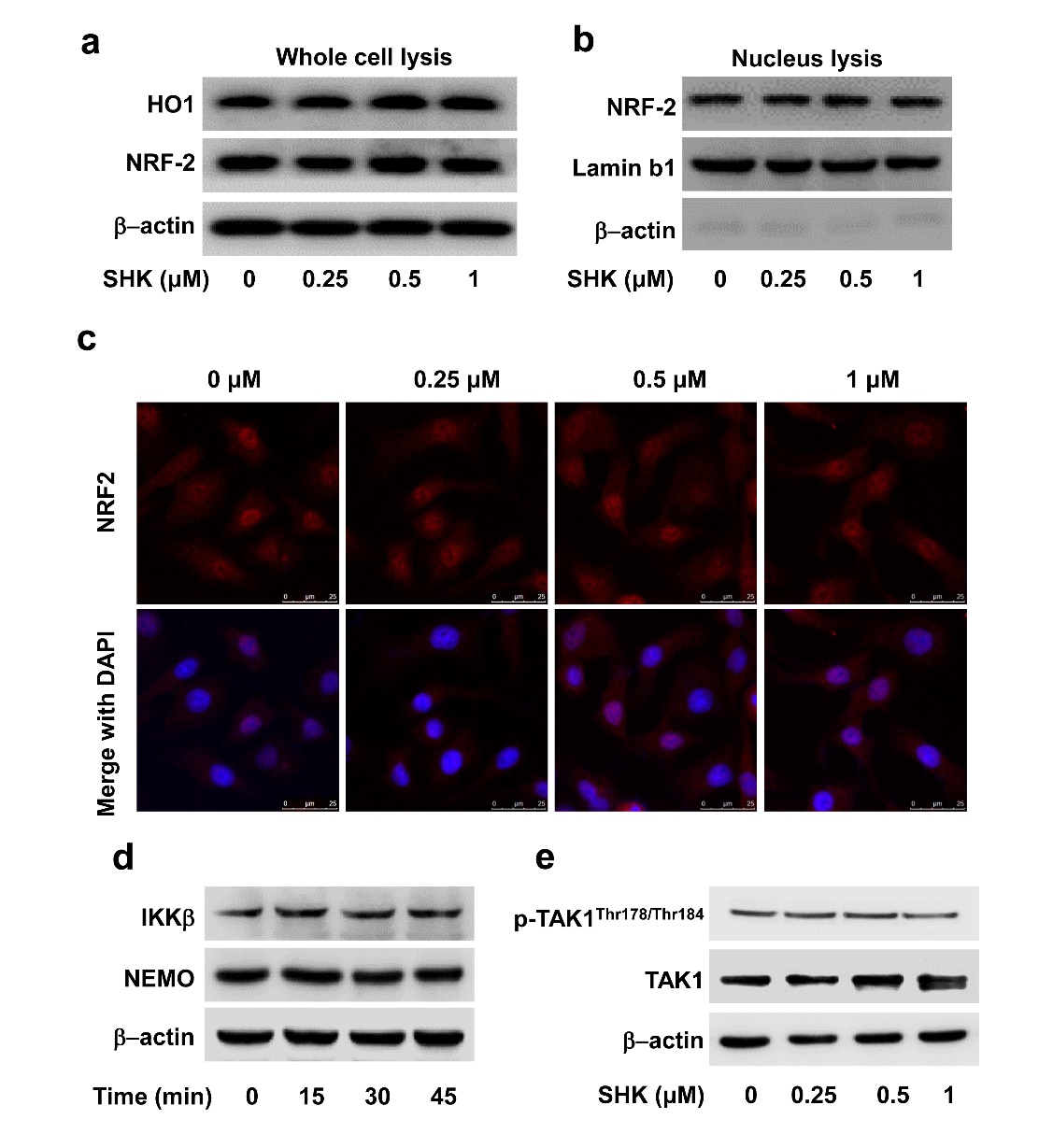
**

**Figure. S6. Effects of SHK on Nrf2/HO-1 signaling. (a)** The expression of Nrf2 and HO-1 was analyzed by western blotting. **(b)** Nuclear extracts were prepared for the western blot analysis of NRF2. Lamin B1 and β-actin were used as controls for sample loading. **(c)** The subcellular localization of NRF2 in LoVo cells following SHK treatment was observed by immunofluorescence staining. **(d)** Cell lysates and SHK (100 μM) for indicate time (15, 30 and 45 min), and then collected the supernatant after high-speed centrifugation. The protein levels of IKKβ and NEMO were analyzed by western blotting. **(e)** The expression of p-TAK1**^Thr178/Thr184^** and TAK1 was analyzed by western blotting.

**
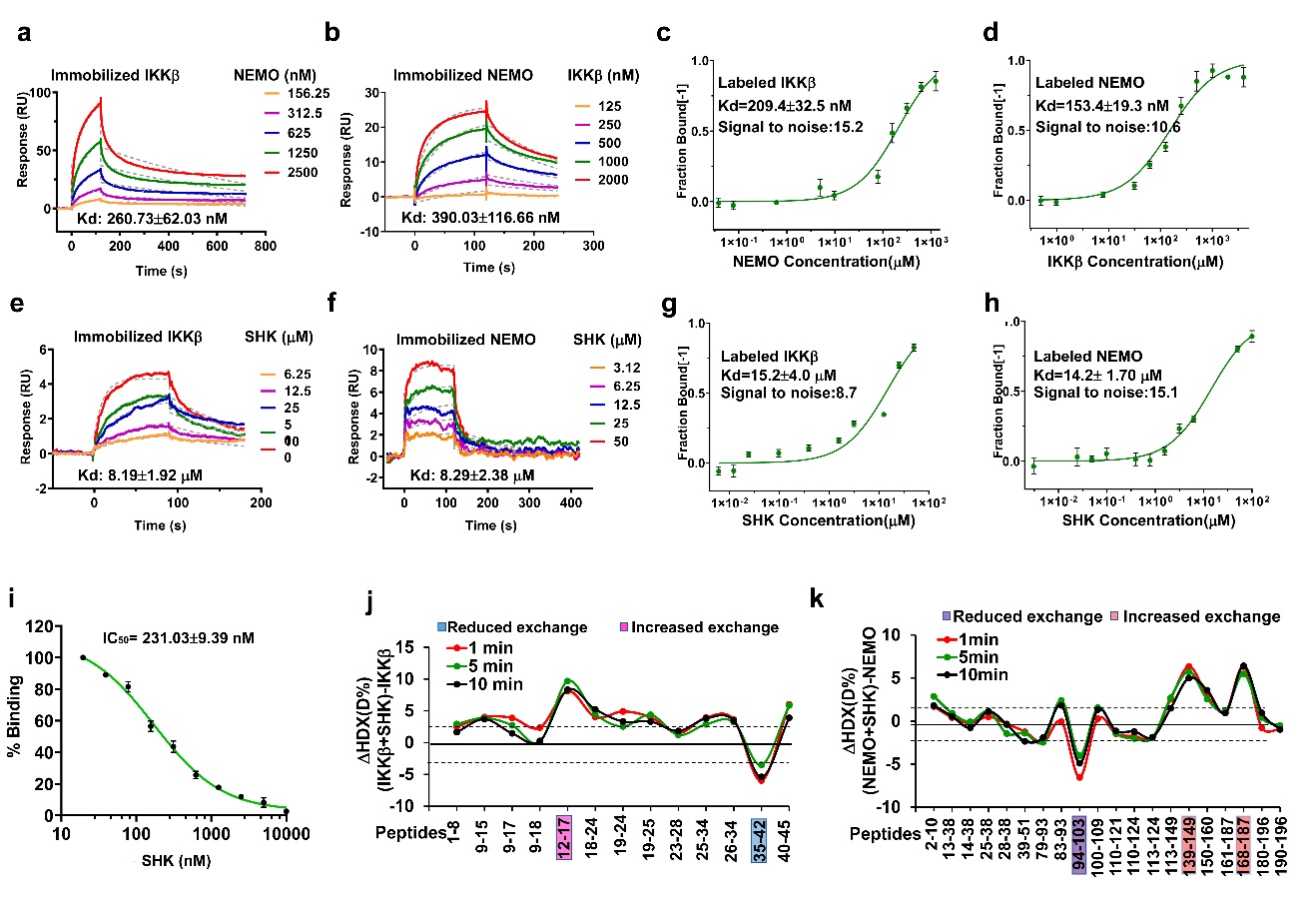
**

**Figure. S7. Effects of SHK on the conformational dynamics of IKKβ or NEMO alone.** **(a, b, e, f)** Measurement of the affinity constant by SPR affinity analysis. The affinity constant (K_D_) values were calculated by global fitting using a steady-state affinity model. Sensor grams were recorded after injection of NEMO (**a**) or SHK (**e**) over MBP- IKKβ immobilized on the chip surface. Sensor grams were recorded after injection of IKKβ (b) or SHK (f) over GST-NEMO immobilized on the chip surface. **(c, d, g, h)** MST-determined binding affinity. Data analysis of the thermophoresis of NEMO **(c)** or SHK **(g)** to IKKβ. Analysis of the thermophoresis of IKKβ (**d**) or SHK (**h**) to NEMO. **(i)** SHK titrated into GST-NEMO_1–196_ bound to biotinylated IKKβ_701-745_. Error bars represent the standard deviation from the average. Comparison of the HDX of IKKβ **(j)** or NEMO **(k)** in the presence and absence of SHK over the measured time points (red-1 min; green-5 min; black-10 min).

**
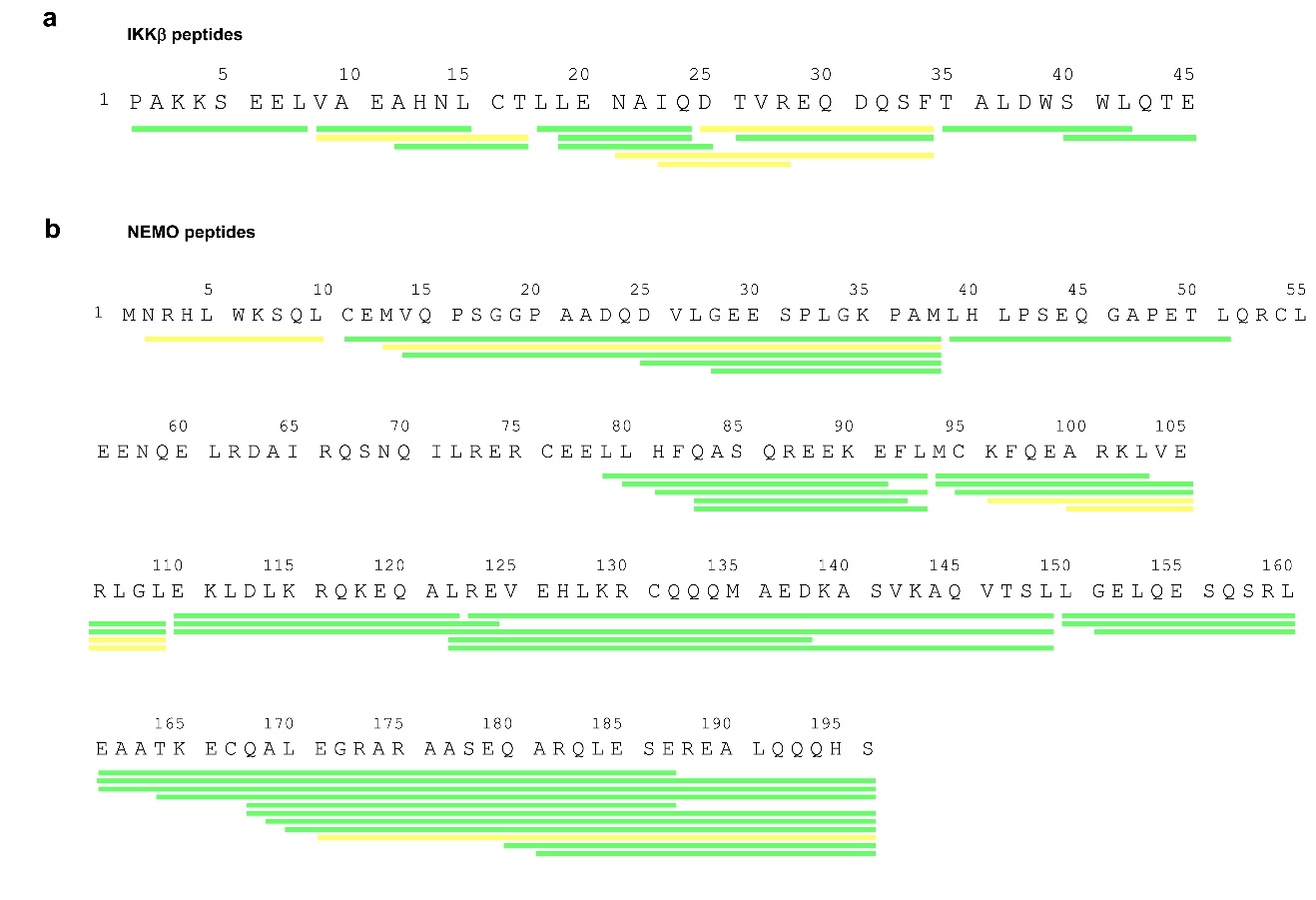
**

**Figure. S8.** HDX-MS peptide coverage map of IKKβ **(a)** and NEMO **(b)**. The coverage was 97.8% **(a)** and 86.7% **(b)**, respectively.

**
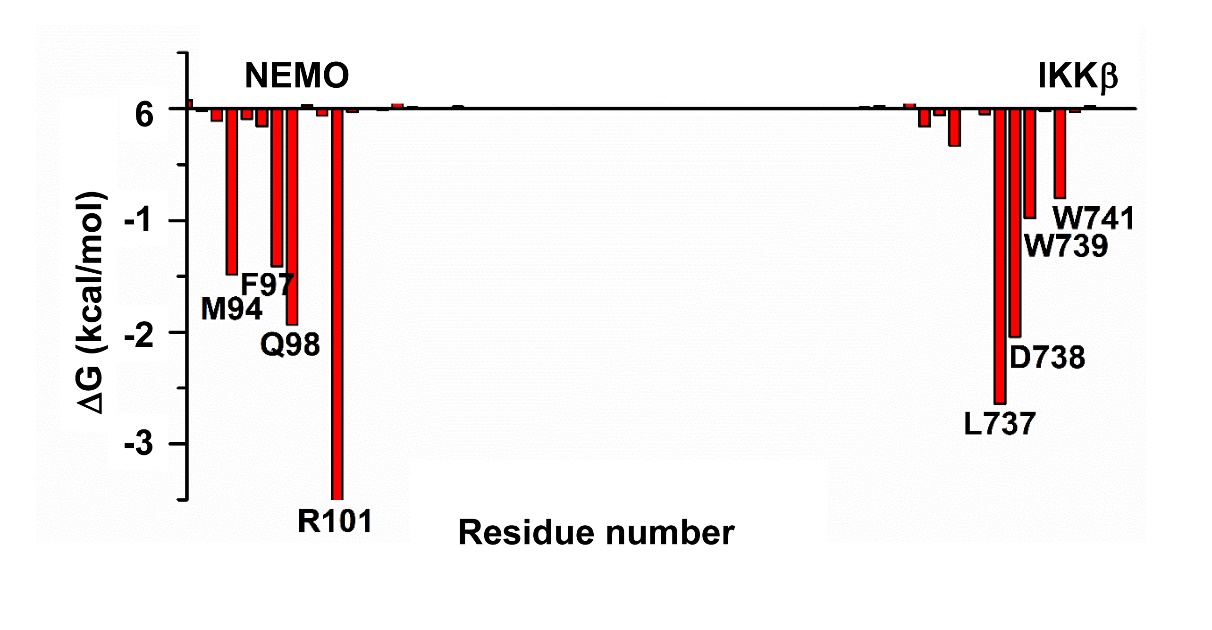
**

**Figure. S9.** **The MM/GBSA of the NEMO/IKKβ/ SHK system.** MM/GBSA free energy decomposition analysis of the total binding free energy per residue for the NEMO/IKKβ/SHK system. The key residues for ligand binding are labeled.

**
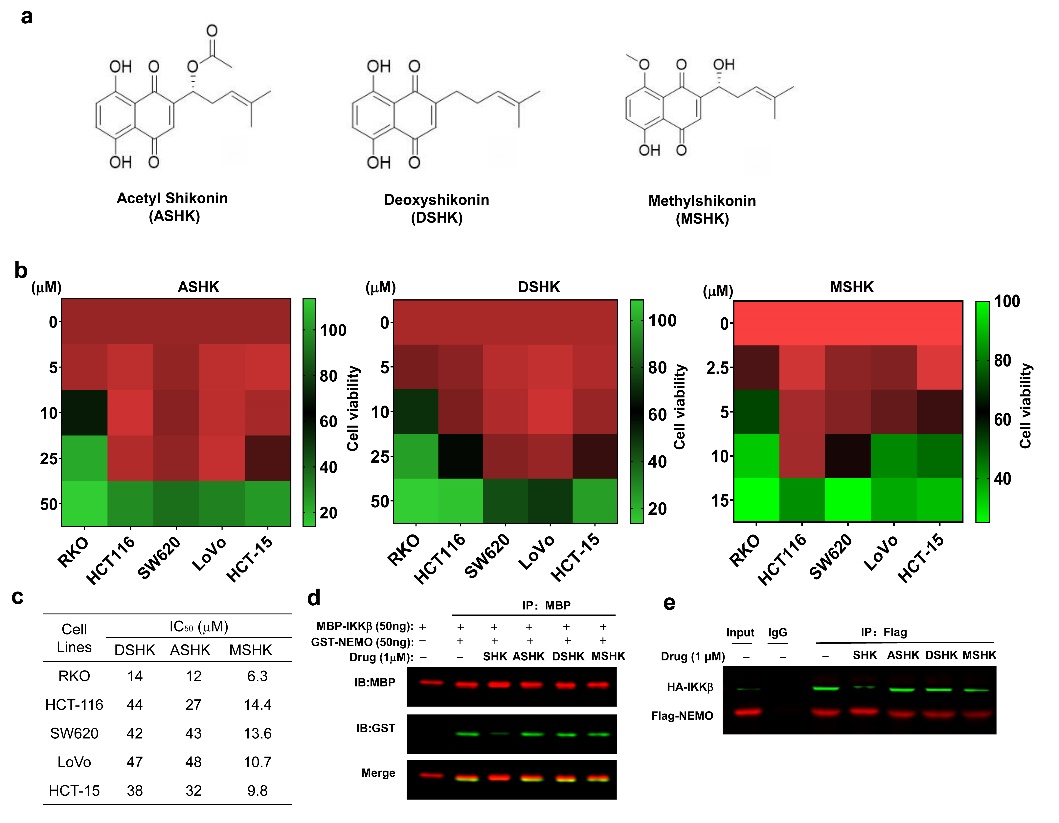
**

**Figure. S10. Effects of SHK derivatives on the stability of IKKβ/NEMO complex. (a)** Chemical structures of acetyl shikonin (ASHK), deoxyshikonin (DSHK) and methylshikonin (MSHK). **(b)** Heatmaps showing the effects of ASHK, DSHK and MSHK on the viability of colorectal cancer cells (LoVo, RKO, HCT-15, HCT-116 and SW620) after treatment for 48h. **(c)** The IC_50_ values of SHK for cell viability inhibition in human colorectal cancer cells (LoVo, RKO, HCT-15, HCT-116 and SW620) were determined. (**d**) Heterologously expressed MBP-IKKβ (701-745) and GST-NEMO (1-196) peptides purified from *E. coli* were pre-incubated to form a stable complex, and then treated with SHK, ASHK, DSHK or MSHK. The interaction of IKKβ/NEMO was analyzed by Co-IP and western blotting using antibodies against MBP and GST. (**e**) HEK293T cells were co-transfected with eukaryotic plasmids encoding full-length Flag-NEMO and HA-IKKβ. Whole-cell extracts were collected and treated with SHK, ASHK, DSHK or MSHK, and analyzed by Co-IP and western blotting using antibodies against Flag and HA.

Table S1 Binding free energy of components of the NEMO/IKKβ/SHK system calculated in MM/GBSA (kcal/mol).

| System | ΔE_vdw_ | ΔE_ele_ | ΔG_GB_ | ΔG_SA_ | TΔS | ΔG_bind_ |
| --- | --- | --- | --- | --- | --- | --- |
| NEMO/IKKβ/SHK | -27.85 ± 3.35 | -19.89 ± 5.06 | 24.47 ± 4.61 | -4.06 ± 0.91 | -12.56 ± 5.75 | -14.77 ± 3.52 |
